# S-Methylmethionine (Vitamin U): A Critical Narrative Review of Pharmacological Mechanisms, Evidence Levels, and Translational Barriers

**DOI:** 10.3390/ph19050743

**Published:** 2026-05-08

**Authors:** Arsen A. Ananian, Tatiana Z. Zelenina, Olga I. Stepanova, Anna A. Popova, Zurab T. Bagatelia, Svetlana I. Kosenkova, Grigory Yu. Evzikov, Boris B. Sysuev, Galina E. Brkich, Natalia V. Pyatigorskaya, Yuriy L. Vasil’ev, Elena O. Bakhrushina

**Affiliations:** 1 A.P. Nelyubin Institute of Pharmacy, First Moscow State Medical University (Sechenov University), 119435 Moscow, Russia; zelenina_t_z@student.sechenov.ru (T.Z.Z.); o.i.nikulina@mail.ru (O.I.S.); popova_a_a_1@staff.sechenov.ru (A.A.P.); kosenkova_s_i@staff.sechenov.ru (S.I.K.); bakhrushina_e_o@staff.sechenov.ru (E.O.B.); 2 N.V. Sklifosovskiy Institute of Clinical Medicine, First Moscow State Medical University (Sechenov University), 119435 Moscow, Russia; bagateliya_z_t@student.sechenov.ru (Z.T.B.); evzikov_g_yu@staff.sechenov.ru (G.Y.E.); vasilev_yu_l@staff.sechenov.ru (Y.L.V.); 3 Faculty of Bioengineering and Bioinformatics, M.V. Lomonosov Moscow State University, 119991 Moscow, Russia; bsb500@yandex.ru; 4 Department of Industrial Pharmacy, First Moscow State Medical University (Sechenov University), 119991 Moscow, Russia; brkich_g_e@staff.sechenov.ru (G.E.B.); pyatigorskaya_n_v@staff.sechenov.ru (N.V.P.); 5 Department of Cellular Systems Engineering, Lomonosov Institute of Fine Technologies, MIREA—Russian Technological University, 125993 Moscow, Russia

**Keywords:** S-methylmethionine, narrative review, vitamin U, cytoprotection, antioxidant activity, tissue regeneration, gastric mucosal protection, methyl donor, preclinical studies, clinical evidence

## Abstract

S-methylmethionine (SMM, also known as vitamin U) is a sulfur-containing vitamin-like compound that has been investigated since the 1940s for its gastroprotective and cytoprotective properties. Historically derived from observations of antiulcer activity in plant-derived foods, SMM has been studied in preclinical models and limited clinical settings for its multilevel pharmacological effects. This narrative review critically evaluates the available evidence on SMM’s pharmacological actions across organ systems, with explicit differentiation between preclinical and clinical data. It covers the most consistently reported gastroprotective and antiulcer effects, as well as antioxidant, anti-inflammatory, cytoprotective, and regenerative activities observed predominantly in preclinical studies. Particular attention is paid to organ-specific protection in the nervous system, liver, kidneys, lungs, skin, eyes, and oral tissues, although human evidence remains scarce. Proposed mechanisms are mediated primarily through suppression of oxidative stress, modulation of inflammatory and immune responses, maintenance of glutathione homeostasis, activation of ERK/NF-κB and Nrf2/Keap1 pathways, and regulation of methylation processes. Building on recent descriptive reviews, this work provides a structured critical synthesis that grades evidence quality, compares SMM with the structurally related methyl donor S-adenosylmethionine (SAMe), and analyses the translational and regulatory barriers that have prevented Western drug registration despite over 70 years of investigation. Despite a substantial preclinical evidence base and historical clinical observations, the level of evidence for many indications remains limited. Well-designed Phase II randomized controlled trials and innovative pharmaceutical formulations (especially topical and mucosal delivery systems) are urgently needed to translate preclinical promise into clinical benefits.

## 1. Introduction

In recent decades, there has been a steady growth of interest in biologically active substances of natural origin that combine cytoprotective and regenerative properties with a favorable safety profile. Such compounds are considered promising candidates both for drug development and for use in nutraceutical products and functional foods, particularly in diseases characterized by mucosal injury and chronic inflammation [1,2].

S-methylmethionine (SMM; also referred to in the literature as methylmethionine sulfonium chloride, MMSC, and “vitamin U”) is a methylated derivative of the essential amino acid methionine and belongs to the class of sulfonium compounds [3]. Historically, interest in SMM arose in the mid-twentieth century due to observations of accelerated healing of gastric ulcerations upon consumption of plant-derived foods rich in this factor, which gave rise to the term “vitamin U” [4]. Throughout this review, ‘SMM’ is used as the primary designation for this compound in all contexts. ‘MMSC’ and ‘methylmethionine sulfonium chloride’ are used only when directly quoting study-specific formulation names. ‘Vitamin U’ appears only in historical context or when referencing dietary/nutraceutical framing. ‘L-MMSC’ designates the L-enantiomer, specifically where stereochemistry is relevant.

Accumulated experimental and clinical data indicate a multidirectional pharmacological profile of SMM [3]. The most thoroughly studied effects of this compound are its gastroprotective and antiulcer actions, which are associated with increased resistance of the gastric mucosa to damaging factors and stimulation of reparative processes [4,5]. At the same time, predominantly preclinical studies have described antioxidant and cytoprotective properties of SMM in several models of toxic and inflammatory organ and tissue injury, including the central nervous system, liver, kidneys, lungs, and ocular structures [6,7,8,9,10]. A separate area of investigation comprises data on the regenerative potential of SMM in skin injuries, where acceleration of wound closure, enhancement of re-epithelialization, and activation of dermal fibroblasts and keratinocytes have been demonstrated [11]. Several experimental studies have described the photoprotective effects of SMM under ultraviolet exposure [12]. Furthermore, isolated reports have emerged on the possible influence of SMM on xenobiotic metabolism systems, which broadens the scope of its potential applications [13].

The contemporary understanding of S-methylmethionine as a metabolically active compound has been consolidated in recent review literature. Kruchinina et al. characterized SMM as a vitamin-like metabolic agent involved in methylation processes, homocysteine metabolism, and antioxidant defense, and noted its functional parallel with S-adenosylmethionine [3]. A near-contemporaneous narrative review by Doba et al. (2025) surveyed gastroprotective, antioxidant, anti-inflammatory, and organoprotective properties of SMM across multiple organ systems [6]. However, neither review provides a structured differentiation of preclinical from clinical evidence levels, a direct mechanistic comparison with S-adenosylmethionine, or a critical analysis of the factors underlying SMM’s absence of regulatory approval in Western jurisdictions—analytical gaps that the present work is specifically designed to address.

At the same time, the concept of “vitamin U” was gradually formed and was initially of a phenomenological nature. In the mid-twentieth century, it was based predominantly on clinical observations indicating a pronounced antiulcer and regenerative effect of raw plant juices, primarily cabbage juice, in patients with peptic ulcer disease of the stomach and duodenum. Along with the work of G. Cheney, subsequent reviews confirmed the reproducibility of the therapeutic effect of dietary therapy in clinical practice; however, the chemical nature of the active principle remained undetermined. In a detailed review of the state of so-called “vitamin U therapy,” Strehler critically analyzed the accumulated clinical and dietological data and concluded that the antiulcer effect of raw plant juices could not be reduced to a single specific compound. The author emphasized that methylmethionine was not identical to the proposed active factor but could be considered a biologically significant cofactor involved in gastric mucosal regeneration. These views reflect a transitional stage in the development of the “vitamin U” concept and anticipate subsequent studies directed toward the chemical identification and pharmacological characterization of S-methylmethionine [4].

Subsequently, the transition from the phenomenological concept of “vitamin U” to a pharmacologically characterized compound was described in detail by Soviet researchers. In the review article by Tumanov and Chekman, SMM was presented as an activated form of methionine with a high methyl potential, supposedly exceeding methionine itself in its capacity for methyl group donation. The authors summarized experimental and clinical data indicating the antiulcer action of the drug, stimulation of regenerative processes in the gastric mucosa, possible reduction of gastric acidity, normalization of hepatic function, and effects on lipid metabolism. The work also highlighted the low toxicity of SMM and discussed biopharmaceutical limitations related to its stability and storage conditions. Thus, this review recorded the transition from the dietary concept of “vitamin U” to the use of S-methylmethionine as an independent pharmacological agent and laid the foundation for its experimental and limited clinical application, as well as further research [5].

Despite its long history of investigation, the evidence base for S-methylmethionine remains heterogeneous. A significant proportion of the available data pertains to early clinical observations and experimental models, while information on certain areas, such as the modulation of detoxification pathways, is limited to isolated studies. This creates a need to systematize the accumulated findings, compare clinical and preclinical data, and identify the key mechanisms of action and limitations of existing studies, which is of fundamental importance for further development of dosage forms and substantiation of clinical indications.

The objective of the present review is to provide a structured critical synthesis of available preclinical and clinical data on the pharmacological effects of S-methylmethionine, with explicit differentiation of evidence levels across organ systems and therapeutic domains. Beyond cataloguing reported effects, this work pursues four specific analytical aims: (1) to characterize the molecular and cellular mechanisms underlying SMM’s pharmacological activities across organ systems; (2) to compare the evidence base and clinical development trajectory of SMM with that of S-adenosylmethionine (SAMe), its structurally related functional analog; (3) to analyze the translational and regulatory barriers that have prevented SMM from achieving drug approval in EU and FDA jurisdictions despite more than 70 years of investigation; and (4) to identify the most mature indications and delivery routes for targeted Phase II clinical research. This multilevel analytical approach distinguishes the present review from prior descriptive summaries and is intended to serve as a practical tool for both clinical researchers and pharmaceutical developers working with SMM.

## 2. Materials and Methods

### 2.1. Literature Search Strategy

A literature search was conducted to identify and systematize clinical and preclinical studies on the pharmacological effects of S-methylmethionine (SMM), also referred to in some sources as methylmethionine sulfonium chloride (MMSC) or vitamin U. The search strategy was designed to encompass both historical publications reflecting the early stage of investigation of this compound and contemporary experimental and clinical works revealing its mechanisms of action.

Electronic literature analysis was performed using the PubMed/MEDLINE, Scopus, Web of Science Core Collection, and Google Scholar databases without date restrictions. Additionally, the reference lists of key articles and review papers were analyzed to identify relevant sources not indexed in the major databases, including publications in regional and Russian language scientific journals. A supplementary search covering the period 2020–2026 was conducted across PubMed, Scopus, and Web of Science to identify recent primary research. This confirmed that fewer than 15 primary research articles on SMM pharmacology have been published globally in this period; these have been incorporated where available (e.g., Egea et al., 2024 [14]; Dong et al., 2026 [15]; Shichkin, 2025 [16]). The predominance of pre-2000 literature reflects the objective historical distribution of SMM research—concentrated in Soviet/Eastern European literature (1970s–1990s) and Japanese/Korean literature (2000s–2010s)—and is discussed as a translational barrier in Section 5.

The following key terms and their combinations were used in search queries: “S-methylmethionine,” “methylmethionine sulfonium chloride,” “vitamin U,” “SMM,” “MMSC” in combination with terms reflecting the main research areas (“gastroprotection,” “anti-ulcer,” “cytoprotection,” “antioxidant,” “anti-inflammatory,” “wound healing,” “skin,” “neuroprotection,” “renal injury,” “radioprotection,” “detoxification,” “xenobiotic metabolism”). To increase search sensitivity, both Boolean operators AND/OR and various transliteration variants and abbreviations encountered in original publications were used.

The review included original experimental studies in vivo and in vitro, clinical studies, clinical observations, case series, and relevant mechanistic studies on the molecular and biochemical effects of SMM. Particular attention was paid to publications evaluating the cytoprotective, antioxidant, regenerative, and metabolic effects of S-methylmethionine, as well as its safety and toxicological profile.

Publication selection was carried out in several stages. In the first stage, titles and abstracts were analyzed. In the second stage, full-text versions of potentially relevant studies were reviewed. In the presence of duplicate publications or overlapping data, preference was given to the most complete and methodologically sound source.

### 2.2. Inclusion and Exclusion Criteria

#### 2.2.1. Inclusion Criteria

Publications satisfying the following conditions were included in the review:

1. Study type
  - original experimental studies in vivo and in vitro;
  - clinical studies (randomized and non-randomized), clinical observations, and case series;
  - mechanistic studies on the molecular, biochemical, and cellular effects of S-methylmethionine.
2. Subject of investigation
  - S-methylmethionine, methylmethionine sulfonium chloride (MMSC), or vitamin U as the primary active component under study;
  - combination drugs and nutraceutical compositions, provided that the contribution of SMM was clearly designated and discussed by the authors. For clarity of mechanistic interpretation, studies are categorized throughout according to intervention type: (A) purified SMM or L-MMSC as sole active agent; (B) MMSC-containing pharmaceutical formulations with defined composition; (C) SMM-containing multicomponent nutraceuticals or combination drugs; (D) plant-derived extracts or juices in which SMM was presumed but not analytically confirmed. Mechanistic conclusions are drawn only from categories A–B; categories C–E are discussed as contextual or hypothesis-generating evidence.
3. Studied effects
  - gastroprotective and antiulcer actions.
  - cytoprotective and antioxidant effects;
  - anti-inflammatory action;
  - regenerative and wound-healing actions.
  - neuroprotective, nephroprotective, hepatoprotective, and pulmonoprotective effects;
  - radioprotective action;
  - effects on metabolism, detoxification, and xenobiotic exchange;
  - safety and toxicological profile data.
4. Publication characteristics
  - articles published in peer-reviewed scientific journals;
  - full-text publications available in English or the Russian language.

#### 2.2.2. Exclusion Criteria

Publications meeting one or more of the following criteria were excluded from the review:

1. studies in which S-methylmethionine was part of a multicomponent composition without discussion of its presumed contribution, mechanism of action, or biological role, making it impossible to interpret the results in the context of the pharmacological effects of SMM;
2. studies devoted exclusively to agrochemical, botanical, or technological aspects of SMM without evaluation of biological or pharmacological effects;
3. review articles, editorials, commentaries, and letters to the editor (used only for reference analysis and identification of primary sources);
4. publications with inadequate descriptions of methodology, absence of control groups, or inability to interpret the results obtained;
5. duplicate publications repeating previously published data without substantial expansion or reanalysis.

### 2.3. Assessment of Evidence Level and Study Quality

The level of evidence and quality of the included studies were assessed considering the heterogeneity of study designs, temporal scope of publications, and nature of the effects of S-methylmethionine under investigation.

Clinical studies were evaluated from the perspective of the evidence hierarchy based on the study design type (randomized controlled trials, double-blind placebo-controlled trials, prospective and retrospective observational studies, and clinical case series). The analysis considered parameters such as the presence of a control group, methods of randomization and blinding, sample size, duration of follow-up, clarity of inclusion and exclusion criteria, and clinical and laboratory endpoints used. Early clinical works from the mid-twentieth century, despite limited correspondence to contemporary standards of evidence-based medicine, were considered in a historical and conceptual context as key sources in the formation of concepts regarding the pharmacological profile of “vitamin U.”

Preclinical studies were analyzed with regard to the adequacy of experimental models, relevance of dosing, routes of administration, duration of exposure, and the set of biochemical, morphological, and molecular parameters assessed. Particular attention was paid to studies in which the effects of S-methylmethionine were confirmed at multiple levels (biochemical, histological, and molecular) and to studies including mechanistic analysis of signaling pathways (e.g., Nrf2/Keap1, ERK1/2, and NF-κB).

Studies in which S-methylmethionine was used as part of combination drugs or nutraceutical compositions were not automatically excluded from the analysis. However, their interpretation was conducted with caution and with mandatory indication of the combination-use context. Priority was given to studies in which the contribution of SMM was discussed by the authors, experimentally substantiated, or logically correlated with the known mechanisms of action of the compound.

The quality of experimental data was assessed with regard to the reproducibility of results, consistency of effects across different models and organ systems, and the presence of dose-dependent effects. Where limitations were present (small sample sizes, absence of functional endpoints, isolated studies on individual research areas), these factors were separately reflected in the review text and discussed in the Section 5.

Overall, given the predominance of preclinical studies and the limited number of contemporary randomized clinical trials, the evidence for certain effects of S-methylmethionine should be considered preliminary. Nevertheless, the consistency of the data obtained, the reproducibility of antioxidant and cytoprotective effects across various experimental models, and the presence of clinical observations and controlled studies in gastroenterological practice allow SMM to be preliminarily considered a biologically active compound with multilevel pharmacological potential, warranting further systematic investigation.

To standardize evidence appraisal, a four-tier classification was applied throughout: Level 1—randomized controlled trials; Level 2—prospective/controlled observational studies or well-designed case series; Level 3—uncontrolled clinical observations or retrospective data; Level 4—preclinical evidence only (in vivo/in vitro). Each pharmacological category in Section 3 is assigned a summary evidence level based on the highest tier of available evidence supporting the primary claimed effect. A consolidated evidence-level summary across all organ systems and indications is provided in Section 5. Evidence levels are additionally summarized in a graphical abstract and in table form.

## 3. SMM Application Experience

The accumulated evidence on the biological activity of S-methylmethionine encompasses a broad spectrum of organs, systems, and pathological conditions. To present this material systematically, the current section follows a two-level organizational framework. The first block (Section 3.1, Section 3.2, Section 3.3 and Section 3.4) covers the effects of SMM on specific organs and systems: the gastrointestinal tract, oral cavity, skin, and central nervous system. The second block (Section 3.5, Section 3.6 and Section 3.7) addresses cross-cutting mechanistic effects—anti-inflammatory, cytoprotective, antioxidant, and metabolic actions—that are not confined to a single organ and, to varying degrees, underlie the effects described in the first block. This approach preserves a clinically oriented, organ-level perspective while avoiding redundant descriptions of similar mechanisms across multiple organ-specific sections.

### 3.1. Digestive System

#### 3.1.1. Preclinical Studies and Experimental Models

The antiulcer and gastroprotective actions of S-methylmethionine are among the earliest and most thoroughly studied pharmacological effects of this compound. A significant contribution to the formation of concepts regarding the mechanisms of action of so-called “vitamin U” was made by Adami and Businco, who investigated the role of histamine in the pathogenesis of gastroduodenal ulcers and the possibility of its pharmacological correction. The authors demonstrated that ulcer formation in patients and experimental models is accompanied by hypergastaminemia, accumulation of histamine in the gastric mucosa, and pronounced edematous-congestive changes in tissues. In experiments on guinea pigs, preliminary administration of derivatives of raw cabbage juice (Brassica oleracea) containing the so-called “U-factor” led to a substantial reduction in the severity of histamine-induced ulcerative damage. Morphological analysis revealed a reduction in edema, normalization of the structure of gastric glands, and enhancement of protective mucous secretion compared to control animals. The authors concluded that the gastroprotective action of the “U-factor” is realized primarily through modulation of vascular-edematous disorders and increased tissue resistance of the gastric mucosa rather than through suppression of acid secretion. Although the chemical nature of the active component has not yet been established in these studies, the data obtained formed the experimental basis for subsequent investigation of S-methylmethionine as one of the key candidates associated with “vitamin U” [17].

A systematic study of the antiulcer activity of S-methylmethionine in experimental models was conducted by Tolstikova, who evaluated the action of the sodium salt of SMM in 13 different rat gastric ulcer models. The compound exhibited both preventive and therapeutic antiulcer actions and prevented and eliminated the ulcerogenic effects of certain drugs [18]. The role of SMM in protecting the duodenal mucosa was additionally confirmed by Salim, who demonstrated that S-methylmethionine provides dose-dependent protection against acute and chronic duodenal ulcers in rats without affecting the level of hyperchlorhydria. These results indicate an important role of antioxidant and cytoprotective mechanisms in the realization of the antiulcer action of SMM [19].

The mechanisms of gastric mucosal healing involving S-methylmethionine were studied in detail by Salim in a series of experimental studies. It was demonstrated that oral administration of methylmethionine sulfonium stimulates healing of ischemia-induced acute gastric mucosal injuries in rats without suppression of acid secretion, indicating a cytoprotective rather than an antisecretory mechanism of action [20]. In subsequent studies, the author demonstrated that SMM accelerates mucosal regeneration in erosive gastritis and chronic gastric ulcers induced by ethanol or reserpine, reducing the area of injury and promoting complete epithelialization of the mucosa [21,22].

A comparative evaluation of the gastroprotective activity of methylmethionine and related compounds was conducted by Nakajima. It was shown that although dimethyl-β-propiothetin surpasses methylmethionine in the magnitude of the preventive effect in stress-induced gastric ulcers in rats, S-methylmethionine itself also demonstrates pronounced protective action without signs of toxicity [23].

The gastroprotective potential of S-methylmethionine has also been confirmed in applied veterinary models. Elbers et al. showed that the addition of SMM to the diet of fattening pigs reduced the incidence of severe esophagogastric erosions and ulcers by approximately 50% compared with that in the control diet [24]. In a later study, Kopinski et al. evaluated the preventive and therapeutic use of SMM in pigs with pre-existing esophagogastric ulcers and found a moderate but statistically significant improvement in the dynamics of ulcerative lesions in animals with a high baseline severity of injury [25].

The molecular mechanisms underlying the cytoprotective action of SMM were studied by Watanabe et al., who demonstrated that SMM protects the gastric mucosa from ethanol-induced injury by increasing the content of surface mucin. The use of the SH-group blocker N-ethylmaleimide established that this effect is mediated by sulfhydryl mechanisms [26]. It was subsequently confirmed that SMM enhances mucin secretion in primary cultures of rabbit gastric mucous cells by stimulating the transport of mucin granules to the apical membrane, and this effect is not associated with the activation of cAMP- or Ca^2+^-dependent signaling pathways [27].

In clinically oriented experimental models, it was also shown that SMM can compensate for the adverse effect of antisecretory therapy on the mucous barrier. Ichikawa et al. demonstrated that combined use of famotidine and SMM prevents the reduction in mucin biosynthesis and accumulation in the gastric mucosa of rats induced by the H_2_-blocker, indicating the potential benefit of combined therapeutic approaches in peptic ulcer disease [28].

Although certain studies extend beyond classical gastroprotection, the data obtained confirm the systemic cytoprotective potential of S-methylmethionine, which is directly relevant to the digestive system organs. Liu et al. showed that SMM serves as a substrate for the BHMT2 enzyme and participates in the protection of the liver from acetaminophen-induced toxicity through regulation of methionine and glutathione metabolism [29]. Similar antioxidant and hepatoprotective effects of SMM have been demonstrated in models of valproate-induced liver injury [8,30], D-galactosamine-induced gastric mucosal injury [31], and aflatoxin-B_1_-induced hepatotoxicity, where activation of the Nrf2/Hmox1 signaling pathway and suppression of inflammatory reactions played a key role [32].

Finally, Cao et al. in a recent study demonstrated that combined supplementation of SMM and antacids in the diet of fattening pigs leads to a reduced degree of keratinization and gastric ulcerative lesions, accompanied by improved growth parameters, underscoring the practical significance of the gastroprotective action of S-methylmethionine under conditions of dietary stress [33].

#### 3.1.2. Clinical Studies and Human Application Data

Historically, the clinical use of S-methylmethionine and the concept of “vitamin U” in gastroenterology were based on studies of plant sources rich in this factor. Early clinical observations were primarily associated with the use of raw plant juices, mainly cabbage juice, which was considered a source of the presumed antiulcer factor.

The work of G. Cheney belongs among the first systematic clinical studies that initiated therapy with so-called “vitamin U” (raw cabbage juice) for peptic ulcer disease. In 1949, in 13 patients receiving 5–12 L of fresh cabbage juice daily, gastric ulcer healing was observed with a mean duration of 6–7 days, which was substantially faster than the previously described period of 10–12 days under standard therapy [34]. Subsequently, Cheney published a series of clinical observations in 100 patients with gastric, duodenal, and jejunal ulcers who received fresh or lyophilized cabbage juice. In 86% of patients, disappearance of the pain syndrome was noted within 4 days, and the mean ulcer healing time was 13–14 days, which differed significantly from approximately 48 days with conventional therapy. Based on these observations, the author emphasized the necessity of conducting controlled clinical studies [35]. In 1956, G. Cheney published the results of a double-blind placebo-controlled study conducted at San Quentin Prison involving 53 patients. Over 3 weeks, the subjects received either concentrated cabbage juice or a placebo. Ulcer healing was documented in 22 patients in the “vitamin U” group compared to seven cases in the placebo group [36]. It should be noted that these studies used plant juices containing a complex of biologically active compounds, and the contribution of S-methylmethionine as a separate component could not be isolated.

These early studies share critical methodological limitations that preclude the attribution of observed effects to SMM specifically: cabbage juice contains numerous bioactive compounds (ascorbic acid, glutamine, flavonoids, and glucosinolates) alongside SMM; neither blinding nor randomization was consistently applied; endpoints were non-standardized; and compound purity and SMM content were not characterized. The risk of publication bias is high, given the single-investigator origin of the primary dataset. These observations should therefore be interpreted as hypothesis-generating rather than controlled evidence of SMM efficacy.

The next stage of clinical research was associated with attempts at pharmacological identification and evaluation of the role of sulfhydryl compounds, including MMSC, as possible active factors in gastroprotective action. In 1992–1993, A.S. Salim investigated the effect of sulfhydryl drugs (DL-cysteine and MMSC) on the healing of gastrointestinal ulcers. In a double-blind placebo-controlled study, patients with relapsing ulcerative colitis received standard therapy (prednisolone and sulfasalazine) alone or in combination with cysteine (200 mg four times daily) or MMSC (500 mg four times daily). After 2 weeks, clinical improvement was observed in 85% of patients receiving sulfhydryl drugs (*p* < 0.01), compared with 51% in the control group. During one year of maintenance therapy, the relapse rate in the cysteine and MMSC groups was approximately 5%, while in the sulfasalazine-only group, it was 27% [37].

In another prospective, double-blind, placebo-controlled study, A.S. Salim evaluated the efficacy of sulfhydryl drugs in stimulating the healing of relapsing duodenal ulcers, as well as their preventive effect in the context of risk factors such as smoking, alcohol consumption, and coffee use. After 8 weeks of therapy, the combination of cimetidine with MMSC or cysteine led to ulcer healing in all observed cases, whereas cimetidine monotherapy was effective in 74% of cases. One year after the completion of treatment, ulcer relapse was noted in 6% of patients in the sulfhydryl drug group and 63% of patients receiving cimetidine alone [38]. In a clinical case series of nine patients with refractory peptic ulcers of the stomach or duodenum, the addition of cysteine or MMSC to standard cimetidine therapy led to ulcer healing within 4 weeks. The author suggested that the gastroprotective effect of sulfhydryl compounds is related to the stimulation of protein synthesis and the formation of protective mucus necessary for the regeneration of the mucosa [39]. In a separate double-blind study of hematemesis treatment caused by erosive gastritis, patients receiving cysteine or MMSC demonstrated more stable hemodynamic parameters and the absence of recurrent bleeding. At 48 h, erosive changes persisted in only 11–12% of patients in the sulfhydryl drug groups compared with 35% in the control group, and severe complications requiring emergency surgical intervention were also significantly less frequently observed in the active treatment groups [40].

More recent clinical studies have evaluated the effects of S-methylmethionine as a separate pharmacological component or as part of combination therapy. In a study by Makhnitska and Babinets involving 45 patients with chronic pancreatitis and *H. pylori*-associated erosive gastritis, the use of a combination of dexpanthenol and S-methylmethionine was accompanied by a statistically significant improvement in the morphological status of the gastric mucosa, reduction in inflammatory infiltration and signs of dysplasia, and more pronounced correction of exocrine pancreatic insufficiency compared with standard therapy [41]. In another study by Drozdov et al., including 37 patients with chronic gastritis, long-term administration of S-methylmethionine at a dose of 300 mg/day led to a reduction in dyspeptic symptoms on the GSRS scale and improvement in quality-of-life indicators on the SF-36 questionnaire, indicating the potential clinical efficacy of S-methylmethionine preparations in functional and inflammatory gastric diseases [42].

Overall evidence level for digestive system application: Level 1 (randomized controlled trials). The distribution of these trials across intervention categories A–E is presented in Table 1.

### 3.2. Oral Cavity

#### 3.2.1. Preclinical Studies and Experimental Models

The potential of S-methylmethionine in protecting oral cavity tissues and organs functionally related to the dental system has been studied in a limited number of experimental studies, primarily in the context of drug-induced oxidative damage. Oktay et al. were the first to investigate the protective properties of SMM in gingival tissue using a rat model of amiodarone-induced toxicity. The authors showed that oral administration of amiodarone was accompanied by pronounced oxidative stress in the gingival tissue, manifested by a significant increase in the levels of lipid peroxidation and sialic acid, as well as a decrease in glutathione content and superoxide dismutase activity. Combined administration of SMM and amiodarone led to the normalization of these parameters, indicating the ability of SMM to reduce oxidative damage and restore the antioxidant status of gingival tissues [43].

Continuing these observations, Alev-Tuzuner et al. evaluated the effect of SMM on parotid salivary glands under conditions similar to those of amiodarone-induced injury in rats. It was shown that seven-day administration of amiodarone caused pronounced biochemical disorders in the parotid gland, including increased lipid peroxidation parameters, myeloperoxidase activity, and inflammatory markers, as well as changes in glycosylation parameters and decreased Na^+^/K^+^-ATPase activity, reflecting impairment of energy and membrane homeostasis in salivary gland cells. Co-administration of SMM effectively neutralized these changes, reducing the severity of oxidative stress, inflammation, and carbohydrate metabolism disorders in salivary gland tissue [44].

#### 3.2.2. Clinical Studies and Human Application Data

The use of S-methylmethionine in dental practice is primarily considered in the context of its local application in inflammatory diseases of periodontal tissues. In a clinical study conducted by Sulym, the efficacy of local application of methylmethionine sulfonium chloride in the form of a gel and therapeutic films on collagen and synthetic bases was evaluated in patients with chronic, generalized periodontitis.

A total of 78 patients with chronic generalized periodontitis of grade I–II severity in the exacerbation phase were included in the study. Patients in the main group (*n* = 47) received treatment using dosage forms containing S-methylmethionine, while those in the control group (*n* = 31) received a traditional treatment regimen. The efficacy of the treatment was assessed based on the dynamics of clinical symptomatology and objective periodontal parameters, including the degree of gingival bleeding, nature and volume of discharge from periodontal pockets, periodontal index, hygiene index, probing depth of periodontal pockets, and radiological examination data. Assessment was conducted immediately after completion of the treatment course and at 1 and 3 months of follow-up.

In the main group, more rapid regression of inflammatory changes was noted, manifested by a reduction in bleeding, densification of the gingival margin, restoration of pale-pink gum coloration, decreased tooth mobility, and reduced depth of periodontal pockets. A pronounced clinical effect was observed in 77% of patients in the main group compared with 51% in the control group. The periodontal index in the main group decreased from 2.82 ± 0.06 to 0.38 ± 0.05 immediately after treatment, while in the control group, it decreased from 2.87 ± 0.06 to 0.82 ± 0.09 (*p* < 0.001). Significant differences between the groups were maintained at the follow-up time points of 1 and 3 months after completion of therapy.

The author also noted good tolerability of S-methylmethionine-based preparations, the absence of recorded adverse effects, and ease of application. It was established that the therapeutic films remain on the surface of the gingival mucosa for 1–3 h, providing prolonged local release of the active substance, which may have clinical significance for maintaining the anti-inflammatory and reparative effect in periodontal diseases [45].

Overall evidence level for oral cavity application: Level 2 (prospective, controlled, observational study).

### 3.3. Regenerative and Dermatoprotective Action of SMM

#### Preclinical Studies and Experimental Models

The regenerative potential of S-methylmethionine has been most thoroughly studied in models of skin injuries, where its active influence on healing processes, cellular proliferation, and tissue remodeling was demonstrated. In contrast to cytoprotective effects realized primarily through reduction of oxidative stress, in the skin, SMM demonstrates the capacity to directly stimulate key cellular mechanisms of regeneration.

A key study in this direction was performed by Kim et al., who demonstrated that topical application of S-methylmethionine accelerates healing of both physical and chemically induced skin wounds in animals. Treatment was accompanied by faster wound surface closure and enhanced re-epithelialization compared to the control groups. In vitro experiments showed that a single exposure to SMM stimulated the proliferation and migration of human dermal fibroblasts, which are key processes for granulation tissue formation and restoration of the dermal matrix. Mechanistic analysis revealed that the regenerative effect of SMM is realized through activation of the ERK1/2 signaling pathway, as pharmacological inhibition of ERK neutralized the stimulatory effect of SMM on fibroblast growth and migration [11].

Continuing these studies, subsequent work from the same research group demonstrated the ability of SMM to exert protective and regenerative actions on the skin under ultraviolet damage conditions. Kim et al. demonstrated that S-methylmethionine increases the survival of keratinocyte progenitor cells and dermal fibroblasts after UVB irradiation, reducing the level of apoptosis and the generation of reactive oxygen species. Simultaneously, activation of ERK signaling, increased collagen synthesis, and decreased matrix metalloproteinase-1 expression were observed, indicating the involvement of SMM in extracellular matrix remodeling processes. Under in vivo conditions, application of SMM before and after UVB irradiation led to a reduction in the severity of skin erythema and prevented depletion of Langerhans cells, indicating a combination of photoprotective and immunomodulatory actions in the skin [12].

Technological aspects of SMM application for the skin were examined by Kim et al., who drew attention to the limited skin penetration of SMM due to its high hydrophilicity. It was demonstrated that the use of penetration enhancers, particularly the combination of oleic acid and ethanol, substantially increases both in vitro and in vivo deposition of SMM in the epidermis and dermis, reaching concentrations sufficient for biological effect realization. These data underscore the importance of rational selection of excipients and dosage forms for effective local application of SMM [46].

Subsequently, the scope of skin regeneration was expanded through the development of modified S-methylmethionine derivatives. Kim et al. synthesized a series of SMMS derivatives lacking the characteristic sulfurous odor of the parent compound and showed that some of them retain or surpass the parent SMM in their ability to stimulate proliferation of dermal fibroblasts and keratinocytes, as well as protect cells from UV-induced damage. These compounds regulated expression of type I collagen and matrix metalloproteinases under ultraviolet stress conditions, making them promising candidates for dermatological and cosmetic products [47].

Additional molecular substantiation of the regenerative potential of S-methylmethionine was obtained by Blium et al., who studied the effect of SMM on chromatin protein methylation processes in rat tissues with different proliferative activities. The authors showed that S-methylmethionine modulates the levels of methylation of histones and non-histone proteins in the heart, liver, small intestinal mucosa, thymus, and spleen. Since chromatin methylation is a key mechanism for regulation of transcription and replication, these results indicate the ability of SMM to affect epigenetic processes underlying cellular proliferation. Although tissue healing parameters were not directly evaluated in this study, the identified effects suggest, speculatively, that epigenetic methylation modulation may contribute to SMM’s proliferative effects; this remains an inferred rather than demonstrated mechanism, as tissue healing was not directly assessed in this study [48].

Additional fundamental information on the possible molecular mechanisms of action of S-methylmethionine was obtained in the study by Areshkina et al., who examined its effect on key enzymes of methyl group metabolism. The authors showed that SMM increases the activity of S-adenosyl-L-homocysteine hydrolase, contributing to selective methylation of the released homocysteine. This leads to a reduction in the inhibitory effect of S-adenosylhomocysteine on methyltransferase reactions and, consequently, to overall activation of methylation processes. Although tissue regeneration parameters were not directly evaluated in this study, the data obtained allow for the consideration of methyl exchange modulation as one of the fundamental biochemical mechanisms underlying the regenerative and cytoprotective effects of S-methylmethionine [49].

It should be noted that most of the data on the regenerative and photoprotective actions of SMM were obtained within a single research school, which underscores the necessity of independent reproduction of these effects in alternative experimental models.

Overall evidence level for regenerative and dermoprotective action: Level 4 (preclinical evidence only).

### 3.4. Psychiatric and Neuropsychiatric Disorders

#### 3.4.1. Clinical Studies and Human Application Data

No preclinical studies investigating the neuropsychiatric effects of S-methylmethionine have been identified in the available literature; the existing evidence is limited to the clinical level.

In one uncontrolled case series, SMM (“vitamin U”) was used in patients with depressive conditions of various nosologies, including manic depressive psychosis, involutional, and vascular depression. The authors reported a reduction in depressive symptoms in patients with different episode durations and prior treatment histories. However, the study employed no control group, no standardized psychometric scales, and no blinding, which substantially limits interpretation and precludes separation of specific pharmacological effects from nonspecific factors. The authors themselves characterized the data as preliminary [50]. This single uncontrolled observation from 1981—with no blinding, no validated psychometric instruments, no control group, and no characterization of SMM dose or purity—provides no reliable clinical evidence for SMM in psychiatric indications and should not be cited as supportive of neuropsychiatric efficacy.

#### 3.4.2. Neuropharmacological Rationale and Mechanistic Context

Despite the absence of controlled clinical data, a mechanistic rationale for investigating SMM in neuropsychiatric contexts can be derived from its established role in one-carbon metabolism. S-methylmethionine functions as a methyl group donor capable of regenerating methionine via betaine-homocysteine methyltransferase 2 (BHMT2), thereby contributing to the S-adenosylmethionine (SAMe) pool. SAMe is a universal methyl donor for a broad range of neurobiologically critical methylation reactions, including the biosynthesis of monoamine neurotransmitters (dopamine, serotonin, and norepinephrine) via catechol-O-methyltransferase, methylation of phosphatidylethanolamine to phosphatidylcholine in neuronal membranes, and regulation of DNA and histone methylation in neural tissue [3]. Depletion of the SAMe pool—clinically associated with major depressive disorder, cognitive decline, and elevated homocysteine—provides a plausible indirect mechanism through which SMM could exert neuromodulatory effects by supporting methylation capacity.

This rationale is further supported by the well-documented clinical efficacy of SAMe in major depressive disorder, where multiple randomized controlled trials have demonstrated antidepressant effects comparable to those of tricyclic antidepressants [3]. Whether SMM contributes meaningfully to central SAMe pools in humans—given its distinct biotransformation profile and organ-specific metabolism—remains unknown and represents a critical mechanistic question for future research. It should be noted that this entire neuropsychiatric rationale is indirect and inferred from SAMe biology; no direct evidence that SMM meaningfully replenishes central SAMe pools in humans currently exists. As noted in Section 4, central SMM bioavailability in humans has not been established, which materially limits the mechanistic plausibility of this rationale. Well-designed, modern randomized placebo-controlled trials employing validated psychometric instruments (e.g., HAM-D or MADRS) in patients with mild-to-moderate depression are needed before any clinical recommendations can be considered.

Overall evidence level for psychiatric and neuropsychiatric disorders: Level 3 (uncontrolled case series).

### 3.5. Anti-Inflammatory Activity

#### 3.5.1. Preclinical Studies and Experimental Models

Anti-inflammatory activity is discussed here as a cross-cutting mechanistic property that underlies many of the organ-specific cytoprotective effects described in Section 3.1, Section 3.2, Section 3.3 and Section 3.6, rather than as a standalone therapeutic indication. The direct preclinical evidence base for SMM as a primary anti-inflammatory agent is limited to a single experimental study and should be interpreted with caution.

The anti-inflammatory activity of S-methylmethionine has been studied in a limited number of preclinical investigations, and according to the available literature, it is essentially represented by a single experimental study. Urazaeva investigated the effect of methylmethionine sulfonium chloride on inflammatory reactions in mice and rats and demonstrated that SMM reduces the permeability of skin capillaries induced by inflammatory stimuli. This effect indicates the ability of SMM to limit the exudative phase of inflammation and stabilize microcirculation.

In the same study, it was established that SMM potentiates the anti-inflammatory action of acetylsalicylic acid. The most pronounced effect was observed when administered one hour before acetylsalicylic acid, suggesting possible pharmacodynamic interaction and a role of SMM in the modification of early stages of the inflammatory response. Simultaneous administration of both compounds resulted in a less pronounced enhancement of the anti-inflammatory effect.

Furthermore, at a dose of 1000 mg/kg, SMM reduced exudation in a rat model of aseptic serositis, confirming its in vivo antiphlogistic activity. Importantly, the anti-inflammatory effect of S-methylmethionine was combined with a gastroprotective action: SMM exerted a protective effect on the gastric mucosa under injury induced by acetylsalicylic acid, which distinguishes it from most nonsteroidal anti-inflammatory drugs [51].

#### 3.5.2. Clinical Studies and Human Application Data

The possibility of using S-methylmethionine in systemic inflammatory diseases has been evaluated in an extremely limited number of clinical studies, and according to the available literature, it is essentially represented by a single clinical study. In a clinical study performed by Starkova, the efficacy of including methylmethionine sulfonium chloride in complex antirheumatic therapy was studied in 34 patients with an active phase of rheumatism, valvular heart disease, and circulatory insufficiency of grade I–II. The control group comprised 20 patients who received standard therapy without the addition of SMM. Patients in the main group received SMM at 50 mg four times daily for 30–40 days.

The activity of the lysosomal enzyme acid phosphatase (AP) in blood serum, considered by the authors as a biochemical marker of the intensity of destructive processes in rheumatism, was used as the primary efficacy parameter. At the beginning of treatment, AP activity in the main group was 24.2 ± 4.2 µkat/L and decreased to 7.8 ± 2.2 µkat/L by the end of therapy (*p* < 0.02), whereas in the control group, no statistically significant dynamics of this parameter were identified. The reduction in AP activity was accompanied by improvement in the clinical picture: pronounced remission was noted in 90.6% of patients in the main group and 65% of patients in the control group, while activation of focal infection was observed in 31% and 40% of patients, respectively. Dynamic determination of AP activity demonstrated its dependence on the severity of the rheumatic process, variant of the disease course, and degree of cardiac insufficiency, with maximum values in the continuously relapsing and prolonged course of rheumatism, as well as in grade II–III circulatory insufficiency. The author interpreted the data obtained as evidence of a more pronounced reduction in destructive processes and improvement in the metabolic state of the myocardium when SMM was included in complex antirheumatic therapy [52].

The obtained results are preliminary in nature and require confirmation in large-scale multicenter randomized controlled trials that evaluate not only biochemical markers (acid phosphatase, AP) but also established clinical endpoints (DAS28, SDAI) and patient-reported quality-of-life outcomes.

Taken together, the anti-inflammatory evidence for S-methylmethionine—comprising one preclinical rodent study and one small uncontrolled clinical series in rheumatic disease—is insufficient to support independent evaluation of SMM as a primary anti-inflammatory therapeutic. Its mechanistic relevance is better understood in the context of the broader cytoprotective and antioxidant profile of SMM: the reduction of NF-κB-mediated inflammatory signaling, suppression of pro-inflammatory cytokines (TNF-α, IL-1β), and modulation of macrophage polarization documented in organ-specific models (Section 3.1, Section 3.2, Section 3.3 and Section 3.6) collectively indicate that anti-inflammatory activity is an integral component of SMM’s cytoprotective mechanism, rather than a discrete pharmacological effect. Modern investigation of SMM’s anti-inflammatory properties would benefit from targeted designs in conditions characterized by mucosal inflammation—such as NSAID-associated gastropathy or chronic periodontal disease—where its combined cytoprotective and anti-inflammatory profile offers the clearest mechanistic rationale.

Overall evidence level for anti-inflammatory activity: Level 3 (uncontrolled clinical observations).

### 3.6. Cytoprotective and Antioxidant Action

#### Preclinical Studies and Experimental Models

The cytoprotective and antioxidant actions of S-methylmethionine have been studied in several preclinical models of acute and chronic injury to various organs, united by the leading role of oxidative stress and inflammatory reactions in pathogenesis.

Mahmarzayeva et al. studied the protective action of SMM in a model of D-galactosamine-induced injury to the brain and cerebellum in rats. It was established that galactosamine administration led to a pronounced decrease in reduced glutathione levels, total antioxidant status, and activity of antioxidant enzymes, including catalase and superoxide dismutase, as well as increased levels of reactive oxygen species, products of oxidative protein damage, and activity of enzymes associated with oxidative stress. Preliminary administration of SMM effectively prevented these changes, restored the antioxidant balance, and reduced the severity of oxidative damage to the brain and cerebellar tissues [53].

The protective action of S-methylmethionine under conditions of convulsive activity was demonstrated by Bayrak et al. in a model of pentylenetetrazol-induced brain injury in rats. Seizure induction was accompanied by increased levels of lipid peroxidation, reactive oxygen species, nitric oxide, and activity of several oxidative and inflammatory enzymes, as well as decreased total antioxidant capacity of brain tissue. Combined administration of S-methylmethionine led to normalization of these parameters, allowing the authors to link the neuroprotective effect of SMM to its pronounced antioxidant properties under conditions of epileptogenic stress [54].

Similar results were obtained by Turkyilmaz in a model of amiodarone-induced brain injury in rats. It was shown that systemic administration of amiodarone causes pronounced oxidative stress in brain tissues, manifested by decreased levels of glutathione, antioxidant enzyme activity, and Na^+^/K^+^-ATPase, as well as increased lipid peroxidation, protein carbonylation, myeloperoxidase activity, and acetylcholinesterase activity. Co-administration of SMM led to reversible restoration of the indicated biochemical parameters, indicating the ability of S-methylmethionine to reduce drug-induced neurotoxic damage [55].

Thus, the neuroprotective effect of SMM in various models is primarily realized through the restoration of antioxidant homeostasis and suppression of inflammatory reactions.

Cytoprotective effects of S-methylmethionine extend to other tissues sensitive to oxidative stress. Antioxidant and cytoprotective action of SMM was demonstrated in a model of drug-induced ocular tissue injury. Tunali et al. studied the effect of S-methylmethionine on lens injury parameters in rats treated with valproic acid. Valproic acid administration was accompanied by a significant increase in lipid peroxidation levels and aldose reductase and sorbitol dehydrogenase activity in lens tissue, alongside decreased reduced glutathione content and activity of antioxidant enzymes, including superoxide dismutase, glutathione peroxidase, glutathione reductase, and glutathione-S-transferase. Combined administration of SMM effectively neutralized the indicated changes, restored the antioxidant status, and reduced the severity of oxidative damage to the lens. These findings indicate pronounced ophthalmological protective activity of SMM, realized through an antioxidant and cytoprotective mechanism of action [10].

In addition to models of direct neurotoxic injury, the cytoprotective action of S-methylmethionine has also been demonstrated under conditions of systemic infectious-inflammatory stress. Kuz’min et al. showed that the administration of S-methylmethionine to guinea pigs with experimental typhoid fever, dysentery, and staphylococcal infection was accompanied by a pronounced decrease in histomorphological damage to internal organs, including the liver, kidneys, spleen, and lungs, compared to that in control animals. The protective effect was observed with both preventive and delayed administration of the drug. In contrast to prednisolone, the use of SMM did not enhance inflammatory-dystrophic changes, indicating its cytoprotective rather than immunosuppressive mechanism of action [56].

A separate area comprises isolated studies devoted to the antitumor and antioxidant actions of SMM, which are currently limited to isolated preclinical investigations. Abouzed et al. conducted the first comprehensive study aimed at evaluating the antioxidant and antitumor activities of methylmethionine sulfonium chloride in a model of hepatocellular carcinoma induced by diethylnitrosamine and carbon tetrachloride in Wistar rats. The authors showed that SMM administration to animals after induction of the tumor process led to significant improvement in biochemical parameters of hepatic function, including levels of aspartate aminotransferase, γ-glutamyl transferase, albumin, globulins, and the albumin/globulin ratio, compared with the untreated hepatocellular carcinoma group. Histopathological examination also revealed a reduction in the severity of structural liver injury during S-methylmethionine treatment. At the molecular level, SMM administration was accompanied by a decreased expression of several markers associated with inflammation, tumor progression, and oxidative stress. In particular, reductions in levels of tumor necrosis factor α (TNF-α), inducible nitric oxide synthase (iNOS), transforming growth factor β1 (TGF-β1), and glypican-3—a marker widely used in the diagnosis of hepatocellular carcinoma—were demonstrated. These changes were combined with a reduction in lipid peroxidation processes, indicating a pronounced antioxidant effect of SMM under conditions of tumor-induced liver injury. The authors concluded that the antitumor action of S-methylmethionine in this model is primarily realized through the reduction of oxidative stress, suppression of inflammatory and immunoregulatory cytokines, and modulation of molecular markers of tumor transformation [57].

The protective potential of SMM is most evident under conditions of extreme oxidative stress, including exposure to ionizing radiation. Antioxidant and cytoprotective action of SMM on renal tissue was demonstrated by Gezginci-Oktayoglu et al. in a model of valproate-induced kidney injury in rats. The authors showed that prolonged administration of valproic acid was accompanied by pronounced histopathological changes in renal tissue, decreased Na^+^/K^+^-ATPase activity, enhanced oxidative stress, inflammatory reaction, and fibrosis. Combined administration of S-methylmethionine led to a significant reduction in malondialdehyde levels and xanthine oxidase activity, and restoration of glutathione content and activity of antioxidant enzymes, including catalase and superoxide dismutase. Furthermore, SMM reduced levels of pro-inflammatory cytokines (TNF-α, IL-1β, MCP-1) and fibrosis markers, including TGF-β and collagen I, indicating a combination of antioxidant, anti-inflammatory, and anti-fibrotic mechanisms of its nephroprotective action [58].

Contemporary data expand the understanding of the cytoprotective potential of S-methylmethionine, demonstrating its ability to modulate chronic inflammation and processes of immune aging (inflammaging). Dong et al. showed that SMM exerts pronounced nephroprotective action in a streptozotocin-induced diabetic nephropathy model in mice. SMM administration dose-dependently improved kidney function parameters and reduced the severity of glomerular hypertrophy, mesangial expansion, and fibrosis. Mechanistic analysis revealed that SMM suppresses macrophage-mediated inflammaging by inhibiting the ERK/NF-κB signaling pathway, reducing pro-inflammatory cytokine production, decreasing the expression of cellular senescence markers, and shifting macrophage polarization toward the reparative phenotype. These data provide preliminary evidence—from a single mouse study—that SMM may modulate macrophage-mediated inflammaging via ERK/NF-κB; however, independent replication is required before this mechanism can be considered established [15].

Cytoprotective action of SMM was also demonstrated in pulmonary tissue under conditions of systemic neurogenic and drug-induced stress. Oktay et al. studied the effect of S-methylmethionine on the condition of the lungs in rats using a model of pentylenetetrazol-induced seizures. The authors showed that convulsive activity was accompanied by pronounced biochemical and cytological changes in pulmonary tissue, including an imbalance of antioxidant enzymes, increased nitric oxide production, and accumulation of inflammatory cells and collagen fibers. Administration of SMM led to a reduction in the severity of oxidative and inflammatory damage to the lungs, allowing the authors to consider it a potential adjunctive agent for pulmonary tissue protection in epileptogenic stress [9].

In a model of drug-induced toxicity, Oztay et al. showed that SMM effectively reduces lung tissue damage caused by valproic acid. Valproate administration led to activation of lipid peroxidation, impairment of antioxidant protection, structural disorganization, and fibrotic changes in the lungs. Combined administration of SMM restored antioxidant enzyme activity, reduced levels of oxidative damage markers, and decreased the severity of fibrosis. Mechanistic analysis indicated the involvement of the Nrf2/Keap1 signaling pathway in the realization of the pulmonoprotective effect of SMM, confirming its antioxidant and cytoprotective properties [59].

Antioxidant and cytoprotective action of S-methylmethionine was also demonstrated under conditions of ionizing radiation. Gessler et al. showed that S-methylmethionine possesses moderate radioprotective activity, providing a reduction in the biological effect of irradiation by 15–30% with a dose reduction factor of approximately 1.2. The proposed mechanism of radioprotective action is associated with a reduction in lipid peroxidation intensity and inhibition of monoamine oxidase activity, indicating the antioxidant and membrane-stabilizing nature of the effect [60].

The role of SMM in the dietary prevention of post-radiation effects was additionally examined in the work of Anistratenko, carried out in the context of the consequences of radiation exposure to the population. The author demonstrated that inclusion of SMM in vitamin complexes and enriched dietary regimens contributed to increased resistance of the organism to ionizing radiation, reduction of post-radiation metabolic disorders, and increased survival in experimental animals. The radioprotective effect of SMM was considered primarily as a consequence of its antioxidant activity and synergism with vitamin E, directed at stabilization of cell membranes and maintenance of oxidative-reductive homeostasis of the organism [61].

Despite the reproducibility of antioxidant and cytoprotective effects of S-methylmethionine in various experimental models, it should be noted that most of the data presented were obtained under conditions of acute toxicity or stress-induced injuries. The heterogeneity of models, dosing regimens, and experimental designs limits direct extrapolation of results and underscores the necessity of further mechanistic and translational studies to clarify the organ specificity and clinical relevance of the identified effects.

To systematize data on the cytoprotective and antioxidant effects of S-methylmethionine, the main preclinical studies and their key results are presented in Table 2.

Overall evidence level for anti-inflammatory activity: Level 4 (preclinical evidence only).

### 3.7. Metabolic Effects

#### 3.7.1. Preclinical Studies and Experimental Models

Preclinical studies have indicated the involvement of S-methylmethionine in the regulation of lipid, energy, and one-carbon metabolism in various experimental models. The most thoroughly studied aspect is the effect of SMM on the lipid profile and cholesterol elimination pathways.

In a series of experimental studies, Seri et al. demonstrated that repeated oral administration of SMM exerts a hypolipidemic effect in various models. In a model of aminoglycoside-induced nephrotic syndrome in rats, administration of SMM at a dose of 1000 mg/kg/day led to a reduction in plasma cholesterol and phospholipid levels and was also accompanied by an improvement in clinical manifestations of nephrotic syndrome, including increased diuresis and decreased proteinuria [7]. In subsequent mechanistic studies, the same authors demonstrated that the hypocholesterolemic action of SMM was not related to suppression of intestinal cholesterol absorption or inhibition of its endogenous synthesis. It was shown that SMM enhances fecal excretion of bile acids and neutral sterols, indicating activation of cholesterol elimination pathways [62]. It was additionally established that SMM dose-dependently reduces elevated levels of low-density lipoproteins and normalizes reduced levels of high-density lipoproteins in a model of dietary hyperlipidemia in rats [63]. A comprehensive evaluation of the hypolipidemic activity of SMM in experiments on rats and rabbits confirmed its ability to normalize diet-induced hyperlipidemia by reducing levels of total cholesterol, β-lipoproteins, and phospholipids in the absence of a significant effect on triglyceride concentrations; the anti-atherogenic effect of SMM was also confirmed histopathologically in a rabbit atherosclerosis model. The activity profile of SMM differed substantially from that of clofibrate, indicating a different mechanism of hypolipidemic action of SMM [64].

Potential enzymatic mechanisms of the hypolipidemic action of SMM were proposed by Matsuo et al., who identified increased formation of 7α-hydroxylated cholesterol metabolites in hepatic microsomal fractions of mice after preliminary SMM administration. The authors interpreted these results as possible evidence of activation of cholesterol-7α-hydroxylase—a key enzyme in cholesterol catabolism [65]. In another study by Matsuo et al., SMM and methionine demonstrated fundamentally different effects on lipid metabolism: in a model of choline-deficient fatty liver disease in rats, SMM normalized the morphological and functional signs of hepatic steatosis, while methionine aggravated fatty infiltration and increased liver mass [66].

The metabolic role of SMM as a methyl group donor was studied by Augspurger et al. in a nutritional experiment on chicks. Researchers have found that dietary SMM possesses pronounced choline-sparing activity and can support growth and metabolic functions in animals with choline and/or methionine deficiency, indicating the involvement of SMM in the regulation of one-carbon metabolism [67].

Individual studies have indicated the potential effects of SMM on carbohydrate and fat metabolism in metabolic disorders. In the study by Matsumoto et al., it was shown that a complex of zinc with SMM, vitamin C, and L-carnitine exhibits insulin-mimetic activity in a rat model of metabolic syndrome, leading to a reduction in visceral fat mass and improvement in blood rheological properties. However, the use of a multicomponent complex does not allow unambiguous attribution of the observed effects exclusively to SMM [68].

The anti-adipogenic action of SMM at the cellular level was demonstrated by Lee et al. in a 3T3-L1 preadipocyte culture. The authors showed that SMM dose-dependently reduces triglyceride accumulation, glycerol-3-phosphate dehydrogenase activity, and expression of key transcription factors of adipogenesis, including PPAR-γ and C/EBP-α, while simultaneously enhancing AMP-activated protein kinase activity. Changes in apoptosis-associated markers were not statistically significant [69].

Contemporary in vivo data were presented by Egea et al., who investigated the effect of SMM in C57BL/6J mice fed a high-fat diet. SMM supplementation led to improvement of glucose metabolism parameters, reducing fasting glucose levels, insulin, and the HOMA-IR index compared with the high-fat diet group without SMM. Gene expression analysis of the liver indicated the regulation of pathways associated with xenobiotic metabolism, glucose metabolism, and circadian rhythms [14].

Given the substantial number of experimental models, differences in study designs, and dosages used, for a clear comparison of the main effects of S-methylmethionine in metabolic disorders, the preclinical study data are summarized in Table 3.

#### 3.7.2. Clinical Studies and Human Application Data

Clinical studies on SMM in the context of metabolic disorders cover various aspects of metabolism, including the regulation of the lipid profile, antioxidant protection, and one-carbon metabolic processes. Overall, the data in this area are limited and are represented by individual clinical studies and observational series.

In one clinical study, the efficacy of the L-form of S-methylmethionine sulfonium chloride (L-MMSC) was evaluated in 26 patients with hypercholesterolemia following oral administration for 8 weeks at a daily dose of 1500 mg. The study revealed a statistically significant reduction in serum total cholesterol by 9.7% (*p* < 0.001) in the absence of significant changes in triglyceride concentrations. Simultaneously, a statistically significant increase in high-density lipoprotein cholesterol (HDL-C) levels was noted, as well as increases in the HDL-C/(total cholesterol − HDL-C) and HDL-C/(total cholesterol + triglycerides) ratios, indicating potentially favorable changes in the lipid profiles. The authors also noted that the therapeutic effect was more pronounced in hospitalized patients than in outpatients [70].

Another aspect of the metabolic action of S-methylmethionine is the regulation of antioxidant protection and detoxification processes. Ataliev et al. studied the state of the glutathione system in healthy individuals and patients with various forms of gastroduodenal pathology, including bleeding ulcers and the early postoperative period. It was shown that complicated peptic ulcer disease and surgical intervention are accompanied by pronounced depletion of the glutathione system, the degree of which correlates with the volume of blood loss and severity of the patient’s condition. The inclusion of the combination drug Kobavit (SMM in combination with glutamic acid) in complex therapy contributed to the restoration of reduced glutathione levels, increased activity of glutathione transferase and glutathione reductase, and activation of detoxification and biosynthetic processes. The authors interpreted these effects as a manifestation of metabolic and antioxidant support of the organism under conditions of pronounced systemic stress [71].

The role of S-methylmethionine as a methyl group donor serves as the basis for its use as a metabolic corrector in patients with cancer. The study by Tarutinov et al. demonstrated that administration of SMM at a dose of 1 g three times per day to patients with esophageal and gastric cancer contributed to the normalization of catecholamine methylation processes, which were disturbed in this pathology. These changes were accompanied by improved hemodynamic stability in the postoperative period, reduced frequency of hypotension episodes and cardiovascular complications, and positive dynamics in certain immunological parameters [72].

Additional data on the metabolic and immunomodulatory potential of S-methylmethionine in patients with lung cancer were presented by Umanskiĭ et al. The authors demonstrated that drug administration was associated with normalization of adenosine metabolism in immunocompetent cells, manifested by increased adenosine deaminase activity and decreased 5′-nucleotidase activity. These changes were accompanied by increased functional activity of natural killer cells and are considered a manifestation of restoration of antitumor resistance through metabolic correction, rather than as direct antitumor action of S-methylmethionine [73].

The clinical studies presented demonstrate heterogeneity in terms of study design, population, and evaluated parameters, which makes direct comparison of results difficult. For a clear comparison of key characteristics and main effects of S-methylmethionine in clinical studies devoted to metabolic disorders, the data are summarized in Table 4.

Overall evidence level for metabolic effects: Level 3 (uncontrolled clinical observations or retrospective data).

## 4. SMM Pharmacokinetics and Biotransformation

The available pharmacokinetic data for SMM, although limited to experimental models and early human studies, permit partial ADME characterization. Oral absorption is rapid in both rats and humans, with predominant distribution in the liver and kidneys [74]. No formal human plasma half-life data are available. Blood-brain barrier penetration has not been directly demonstrated for SMM; the neuroprotective effects described in Section 3.4 and Section 3.6 may therefore reflect peripheral antioxidant and metabolic effects rather than direct central action—a critical mechanistic uncertainty that must be resolved before neurological indications can be considered clinically viable. Topical dermal penetration is low owing to SMM’s hydrophilic sulfonium structure; penetration enhancers (oleic acid/ethanol combinations) have been shown to substantially increase epidermal and dermal deposition in animal models [46]. To date, no standardized human Phase I pharmacokinetic study with a defined pharmaceutical formulation has been conducted.

The potential for pharmacokinetic and pharmacodynamic interactions between SMM and co-administered agents has not been formally investigated. Mechanistically, three interaction categories warrant consideration. First, as a methyl group donor participating in homocysteine remethylation, SMM may interact additively or synergistically with other methyl donors (betaine, SAMe, folate, and vitamin B12), potentially causing supraphysiological methylation flux; this is speculative and unconfirmed in human studies. Second, the induction of CYP2A6 observed in vitro [13] raises the theoretical possibility of altered metabolism of CYP2A6 substrates (e.g., nicotine, cotinine, and certain anticonvulsants), although its in vivo relevance is unknown. Third, given the glutathione-restoring and antioxidant properties of SMM, additive effects with N-acetylcysteine or glutathione precursors cannot be ruled out. No clinical DDI data exist; these interactions remain hypothetical, pending dedicated pharmacological investigation.

Understanding the pharmacokinetic profile of S-methylmethionine is essential for interpreting its biological effects: systemic bioavailability, biotransformation pathways, and the organ-specificity of its metabolism largely determine where and how the compound exerts its actions. Despite the limited number of studies dedicated to this topic, the accumulated data allow a coherent picture of the fate of SMM in the organism to be drawn. Among the earliest investigations aimed at characterizing the metabolic pathways of S-methylmethionine was the work of Bezzubov and Gessler, in which gas–liquid and column liquid chromatography were used to characterize the biotransformation routes of S-methylmethionine in humans and experimental animals. The authors demonstrated that SMM undergoes biotransformation without signs of excessive tissue accumulation and can be reliably identified in biological media, indicating its systemic availability and a predictable metabolic profile. These data laid the foundation for subsequent studies on the pharmacokinetics and biological actions of SMM [75].

Further development of pharmacokinetic concepts of “vitamin U” was presented in the work of the same group, Gessler and Bezzubov, who studied the absorption, distribution, and assimilation of S-methylmethionine in rats and humans. According to the authors, SMM is characterized by rapid absorption and active incorporation into the metabolism of the organism, with predominant accumulation in the liver and kidney. The main pharmacokinetic parameters of various optical forms of S-methylmethionine were determined, showing that the L-form is predominantly utilized by the organism, while combined administration of D- and L-forms stimulates the assimilation of the D-isomer. These results indicate the stereospecificity of SMM metabolism and confirm its systemic bioavailability in both experimental animals and humans [74].

An important aspect of the pharmacokinetic profile of S-methylmethionine is its stability and bioavailability when consumed with food. Lee et al. studied the digestive stability and bioaccessibility of SMM from kimchi cabbage using a simulated in vitro system reproducing successive stages of human digestion. It was shown that SMM is characterized by high stability in the acid environment of the stomach and does not undergo significant degradation under the influence of energy load or food matrix. At the same time, the bioaccessibility of SMM from a plant source substantially exceeded that of the pure substance and varied within 8.8–14.7% at different stages of digestion. The data obtained indicate that the natural food matrix can contribute to the preservation and release of S-methylmethionine in the gastrointestinal tract, which is of fundamental importance for its nutraceutical application and may partly explain the effectiveness of plant sources of SMM in early clinical observations [76].

Additional information on the biotransformation of S-methylmethionine was obtained in a later study by the same research group. Gessler et al. studied the metabolism of SMM in rats and showed that its biotransformation actively proceeds in the liver, kidneys, and digestive tract organs. It was established that in the liver and kidneys, SMM metabolism is realized through two main pathways: through remethylation of homocysteine with formation of methionine, as well as through enzymatic hydrolysis with formation of dimethylsulfide and homoserine. In contrast, the predominant activity of S-methylmethionine sulfonium hydrolase was identified in the gastrointestinal tract. The data obtained indicate the organ specificity of SMM metabolism and confirm its involvement in the regulation of one-carbon and sulfur-containing metabolism, which potentially may be important for the realization of its systemic cytoprotective and detoxification effects [77].

In recent years, studies have expanded our understanding of the role of S-methylmethionine in the regulation of xenobiotic metabolism systems. Choi et al. studied the effect of SMM in graviola leaf extracts on intestinal bioavailability and nicotine detoxification in experimental in vitro models of intestinal and hepatic epithelial cell lines. Thermal processing of plant materials and enrichment of the extract with auxiliary plant components (kale extract) led to increased cellular uptake of SMM in a Caco-2 intestinal epithelium model. Functionally, this was accompanied by enhanced nicotine metabolism in HepG2 cells, as evidenced by increased cotinine formation and significant induction of the CYP2A6 isoenzyme. The data obtained indicate the ability of S-methylmethionine to modulate enzymatic systems of xenobiotic biotransformation and underscore its potential role as a nutraceutical component contributing to detoxification with increased bioavailability [13]. It should be emphasized that the identified effects were obtained in vitro and using plant extracts, which limits the direct extrapolation of results to isolated S-methylmethionine in vivo.

Despite its satisfactory oral bioavailability in rodents and humans, S-methylmethionine exhibits low skin permeability owing to its highly hydrophilic sulfonium structure. No contemporary human Phase I pharmacokinetic studies with standardized formulations have been performed, and the stereospecific metabolism of the L- versus D-isomer remains insufficiently explored in clinical settings, which is a gap with direct practical consequences. The clinical relevance of SMM stereochemistry is not merely theoretical: among the registered medicinal products in Japan, Korea, Taiwan, and Hong Kong, the active substance is specified as the L-form (L-MMSC) in the majority of registration dossiers, reflecting a regulatory and pharmaceutical preference for the naturally occurring enantiomer [78,79,80,81,82,83,84]. The pharmacokinetic data available from Gessler and Bezzubov indicate that the L-form is preferentially utilized in vivo, while co-administration of D- and L-forms may stimulate D-isomer assimilation through as-yet undefined mechanisms [74]. Whether the D-isomer contributes to, is neutral toward, or potentially antagonizes the pharmacological effects of L-SMM at therapeutic doses has not yet been established in human subjects. Given that bulk pharmaceutical SMM may be supplied as a racemic mixture depending on the synthetic route, and that racemate vs. enantiopure compositions may differ in both efficacy and safety profiles, resolution of this question is a prerequisite for rational pharmaceutical development—particularly for novel topical and mucosal delivery systems, where local tissue concentrations and stereoselective biotransformation may differ substantially from systemic oral exposure.

Taken together, the available data indicate that S-methylmethionine is characterized by satisfactory bioavailability, a predictable metabolic profile, and organ-specific biotransformation effects. The stereospecificity of its metabolism, organ tropism of its distribution, and involvement in the regulation of one-carbon metabolism provide the pharmacokinetic basis for the systemic effects of SMM described in Section 3. Nevertheless, existing pharmacokinetic data have been predominantly obtained from experimental models, and no standardized clinical pharmacokinetic studies have been conducted to date. This substantially limits the possibilities for rational dosing and predicting inter-individual variability in therapeutic response.

### Drug Delivery Systems and Formulation Approaches

Several delivery strategies have been explored to address SMM’s key biopharmaceutical limitations: hydrophilicity, instability, and organoleptic properties. For topical dermal application, a combination of oleic acid and ethanol as penetration enhancers substantially increased SMM deposition in the epidermis and dermis in animal models [46]. For mucosal application in the oral cavity, MMSC-containing therapeutic films on collagen and synthetic bases provided prolonged local release (1–3 h) with favorable clinical tolerability in a periodontal study [45]. Semi-solid formulations (gels and films) are currently the most pharmaceutically mature platforms for SMM. At the preclinical level, synthesis of SMMS derivatives lacking the characteristic sulfurous odor while retaining or exceeding SMM’s biological activity has been demonstrated, expanding the candidate pool for cosmetic and dermatological products [47]. Systemic oral delivery in Japan and Asia utilizes tablet and granule forms of L-MMSC [78,79,80,81,82,83,84]. To date, no mucoadhesive gastric or intestinal formulations with controlled release have been described in the primary literature, representing a key pharmaceutical gap given the documented instability of SMM in solid oral dosage forms.

## 5. Discussion

In accordance with the objective of the present review, an analysis and critical comparison of available clinical and preclinical data on the pharmacological effects of S-methylmethionine was conducted. The reviewed results confirm that the most substantiated and studied area of SMM application remains gastroprotection, while data on its cytoprotective, antioxidant, and regenerative effects are predominantly based on experimental models and require further validation. Simultaneously, the identified limitations of the evidence base and biopharmaceutical characteristics of SMM underscore the need to develop optimized delivery systems and conduct well-designed clinical studies to substantiate potential indications for use.

**Figure 1:**
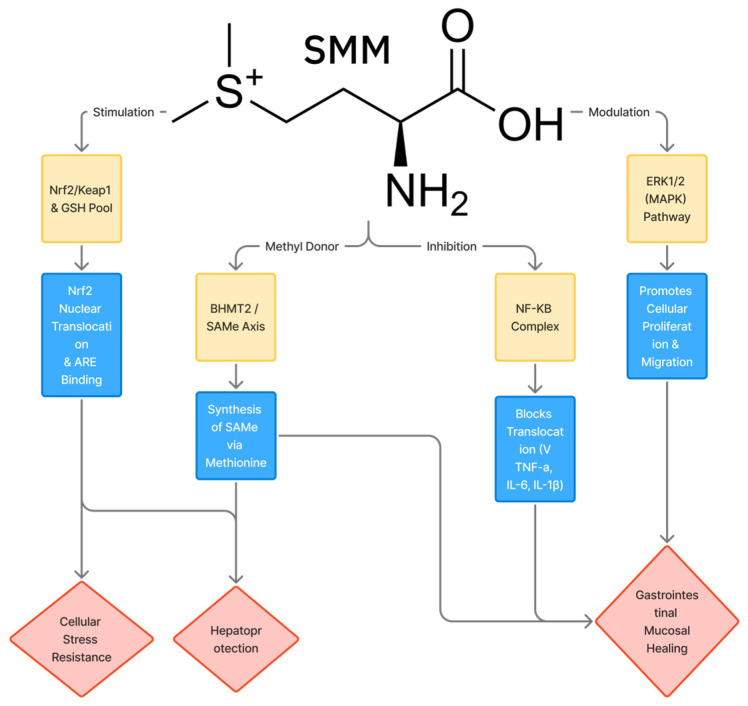
SMM’s primary molecular targets. Arrows indicate stimulatory pathways, molecular transitions, or inhibitory effects (e.g., SMM-mediated suppression of NF-κB nuclear translocation).

Figure 1 presents a mechanistic pathway diagram integrating SMM’s primary molecular targets (Nrf2/Keap1, ERK1/2, NF-κB, BHMT2/SAMe axis, glutathione homeostasis). Table 5 summarizes the highest evidence levels achieved for each indication. Gastroprotection (Level 1–2), periodontal application (Level 2), and hypolipidemic effects (Level 2) were the most clinically advanced areas. Neuropsychiatric, radioprotective, and systemic anti-inflammatory indications currently achieve Level 3–4 only.

The present review systematizes disparate clinical and preclinical data on the pharmacological properties of SMM, allowing it to be discussed not only in the context of gastroprotection but also as a vitamin-like compound with potentially multidirectional biological activity. Analysis of the included studies shows that the described effects of SMM are realized at various levels—from molecular and cellular mechanisms to organ-specific and systemic tissue protection—while the degree of their clinical validation varies substantially.

One of the most consistently reproducible effects of S-methylmethionine is its gastroprotective effect. Clinical data from the mid-twentieth century, including controlled studies by G. Cheney and A.S. Salim, demonstrated acceleration of gastric and duodenal ulcer healing, reduced relapse frequency, and decreased risk of complications when SMM was added to standard therapy. Contemporary preclinical studies have supplemented these observations with mechanistic data indicating the key role of antioxidant and sulfhydryl (SH-dependent) mechanisms, stimulation of mucin secretion, and enhancement of mucosal regeneration without affecting acid secretion. Such a profile distinguishes SMM from classical antisecretory agents and underscores its significance as a cytoprotective rather than a symptomatic agent.

The relevance of gastroprotective and reparative approaches to gastrointestinal disease therapy has also been emphasized in contemporary reviews. In particular, in the work by Shichkin [16], S-methylmethionine is considered a key nutrient acting not only on aggressive factors (acidity, inflammation) but also on the protective and reparative mechanisms of the mucosa, including epithelial regeneration, mucin synthesis, and maintenance of cellular metabolism. The author emphasizes that standard antisecretory and eradication therapy regimens often do not provide complete mucosal restoration, which substantiates the feasibility of including cytoprotective and regenerative components, such as SMM, as adjunctive therapy. Thus, contemporary concepts of the treatment of erosive-inflammatory gastrointestinal lesions are consistent with the accumulated clinical and experimental data on SMM-mediated mechanisms of mucosal protection and repair [16].

Beyond the gastroenterological context, the systemic character of the action of S-methylmethionine has been confirmed by preclinical study data in metabolic disorder models. It was shown that SMM exerts hypolipidemic and anti-atherogenic actions, regulates the lipoprotein profile, enhances cholesterol elimination, and modulates energy metabolism through activation of AMPK-dependent pathways. In contrast to methionine, which aggravates steatotic changes in the liver in several models, SMM demonstrates a metabolically favorable profile, which appears to be associated with its role as a methyl group donor and its involvement in one-carbon metabolism. These properties suggest, at a preclinical level only, that S-methylmethionine warrants further investigation in metabolic disorders and conditions associated with oxidative stress.

Particular attention should be paid to the data on the cytoprotective and antioxidant actions of SMM under conditions of drug-induced and toxic injury to various organs. In models of central nervous system, kidney, lung, and ocular tissue injury, SMM consistently reduced the severity of oxidative stress, normalized antioxidant enzyme activity, suppressed inflammatory reactions, and inhibited fibrotic processes in several cases. Contemporary studies have supplemented classical concepts by showing that SMM can modulate the ERK/NF-κB and Nrf2/Keap1 signaling pathways and reduce macrophage-mediated inflammaging in chronic kidney diseases. This indicates that the cytoprotective action of SMM is realized not only through direct scavenging of free radicals but also through more complex immunomodulation and regulation of inflammatory-fibrotic pathways. It should be noted that pathway-level mechanistic conclusions (ERK/NF-κB, Nrf2/Keap1) are based exclusively on preclinical models and require confirmation in human-tissue systems.

A critical analytical reference point for evaluating SMM’s translational potential is its structural and mechanistic relationship with S-adenosylmethionine (SAMe). Both compounds function as sulfonium-based methyl group donors in one-carbon metabolism and have been investigated for their hepatoprotective, neuroprotective, antidepressant, and antioxidant properties. However, their clinical development trajectories have substantially diverged. SAMe has accumulated a robust clinical evidence base that includes well-powered randomized controlled trials in intrahepatic cholestasis of pregnancy, alcoholic liver disease, major depressive disorder, and osteoarthritis. It holds drug registration status in Germany, Italy, Spain, and several other EU countries and is available as a prescription or regulated OTC agent in multiple jurisdictions. In contrast, SMM lacks equivalent RCT data for any of these indications and remains unregistered as a medicinal product in all Western jurisdictions. This disparity is not primarily pharmacological in origin—both compounds act through overlapping methylation-dependent and antioxidant pathways—but rather reflects structural differences in commercial history, intellectual property positioning, and regulatory strategy. SAMe benefited from decades of industry-sponsored clinical development, whereas SMM did not. Recognizing this distinction is essential for defining realistic translational expectations for SMM and for identifying indication-specific niches—particularly mucosal cytoprotection, topical oral applications, and adjunctive gastroprotection—where it may offer practical advantages, such as superior cost-effectiveness, favorable topical stability in semi-solid formulations, and an established safety record in populations (including pregnant women) for whom SAMe use is less documented. The evidence base for SMM’s metabolic and cytoprotective effects would benefit from a comparison with contemporary literature on structurally related sulfur-containing methyl donors, including trimethylglycine, taurine, and N-acetylcysteine, for which more extensive and recently replicated evidence bases exist [85,86,87,88,89,90].

**Figure 2:**
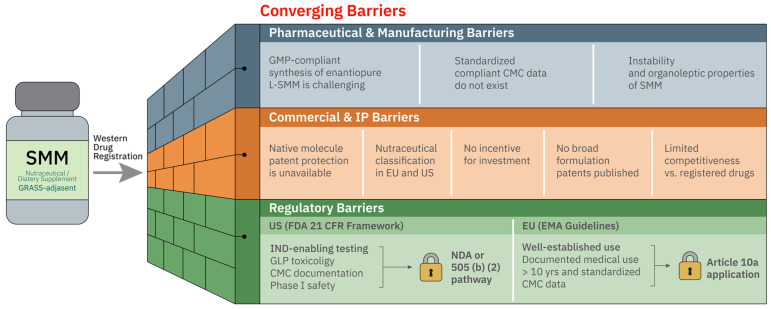
Translational barriers to Western SMM registration.

The persistent absence of Western drug registration reflects converging regulatory, commercial, and pharmaceutical barriers. Under the FDA 21 CFR framework, SMM would require IND-enabling studies (GLP toxicology, CMC documentation, Phase I safety) before any NDA or 505(b)(2) pathway could be pursued; its current GRAS-adjacent supplement status removes the commercial incentive for this investment. Under EMA guidelines, a well-established use application (Article 10a) would require documented medical use exceeding 10 years with recognized efficacy—achievable in principle given the historical record—but would still require standardized CMC data in line with regulatory requirements, which do not currently exist for SMM. GMP-compliant synthesis of enantiopure L-SMM at a pharmaceutical grade represents a non-trivial manufacturing challenge, given SMM’s instability and organoleptic properties. Patent protection for the native molecule is unavailable, and no novel formulation patents of sufficient breadth to incentivize Phase II−III investments have been published. The nutraceutical classification in the EU and US markets, while commercially viable, forecloses reimbursement pathways and limits the compound’s competitiveness against registered alternatives (SAMe, sucralfate, misoprostol) in gastroenterological indications. Overcoming these barriers requires a coordinated strategy: selection of a patentable delivery platform (e.g., mucoadhesive L-SMM gel), a 505(b)(2) or Article 10a regulatory pathway, and a Phase II trial in a single well-defined indication with biomarker-validated endpoints. The concentration of primary SMM research in Soviet/Eastern European and Japanese/Korean literature prior to 2000, combined with the near-absence of contemporary independent replications, constitutes an additional translational barrier: the evidence base lacks the geographic and methodological diversity required by contemporary regulatory agencies for efficacy substantiation. Figure 2 schematizes the translational barriers preventing Western drug registration.

The regenerative potential of S-methylmethionine has been most clearly demonstrated in dermatological models. Unlike many antioxidants that act primarily as “passive” scavenger agents, SMM can actively stimulate cellular proliferation and migration, as confirmed in wound-healing models and dermal fibroblast cultures. Activation of ERK1/2 signaling, enhanced collagen synthesis, and modulation of matrix metalloproteinase activity form the molecular basis of its regenerative action. Additional confirmation is provided by data on the effect of SMM on chromatin methylation processes, suggesting the involvement of epigenetic mechanisms in the realization of its proliferative effects.

In studies addressing neuropsychiatric effects and systemic anti-inflammatory activity, the evidence base remains the weakest and is limited to isolated studies conducted in the 1970s and the 1980s. This highlights the pressing need for modern Phase II–III clinical trials to substantiate these early observations.

The potential of S-methylmethionine in dental practice deserves separate discussion, which, despite the limited number of studies, logically follows from its gastroprotective, cytoprotective, and regenerative properties. The clinical data presented in this review indicate the clinical efficacy of local SMM dosage forms in inflammatory periodontal diseases, manifested by accelerated regression of inflammation, reduction of bleeding, decreased probing depth of periodontal pockets, and prolonged therapeutic effect with good tolerability. Preclinical studies have complemented these observations by demonstrating the pronounced antioxidant and tissue-protective actions of SMM in gingival tissues and salivary glands under drug-induced oxidative stress. These data suggest that S-methylmethionine is a promising active component for the development of dental dosage forms with local action aimed at restoring oral mucosal tissues, modulating the inflammatory response, and stimulating regeneration, which is of particular importance in chronic inflammatory and destructive periodontal diseases.

Additional confirmation of the pharmacological significance of the S-methylmethionine structure is provided by contemporary works that use SMM not as an independent therapeutic agent but as a structural prototype for the development of new bioactive compounds. Thus, Zhang et al. developed a series of sulfonium-containing polypeptides structurally mimicking SMM, which demonstrated ultrahigh antibacterial activity and selectivity, including against methicillin-resistant Staphylococcus aureus (MRSA). Although in this study the biological activity was demonstrated for synthetic polymers rather than for SMM itself, the results obtained underscore the key role of the sulfonium group and high cationic density characteristic of SMM as pharmacophoric elements providing membrane-active and oxidatively mediated mechanisms of action. These data expand understanding of the translational and conceptual potential of S-methylmethionine, viewing it not only as a bioactive compound but also as a promising structural template for rational design of new drugs [91].

Although the gastroprotective effects of S-methylmethionine are supported by historical controlled clinical data, evidence for most organoprotective, regenerative, and metabolic effects remains predominantly preclinical. Key limitations include species differences in one-carbon metabolism and BHMT2 expression, almost complete absence of long-term safety data (>6 months) and dose-response studies in humans, and lack of modern pharmacokinetic–pharmacodynamic investigations. Future research should prioritize adequately powered Phase II–III randomized controlled trials using validated clinical and biomarker endpoints, as well as the development of advanced delivery systems (mucoadhesive gels, films, and penetration-enhanced topical formulations) to overcome the current biopharmaceutical constraints.

An important aspect discussed in the framework of the present review is the safety and limitations of S-methylmethionine application. Toxicological studies have shown the absence of teratogenic action and a favorable reproductive safety profile, which advantageously distinguishes SMM from certain other biologically active compounds found in cruciferous plants. At the same time, SMM is characterized by certain pharmaceutical limitations, including pronounced organoleptic properties, susceptibility to oxidation, and possible thermolability. Contemporary studies on structural modification, derivative development, and dosage form optimization have demonstrated that these disadvantages can be successfully compensated, which is especially relevant for dermatological and nutraceutical applications [92].

The publication of the Doba et al. narrative review, covering the gastroprotective, antioxidant, anti-inflammatory, and organoprotective effects of SMM across multiple organ systems, warrants direct and transparent acknowledgment. The conclusions of that review are broadly consistent with those of the present work, and both confirm the multilevel pharmacological potential of the compound at low toxicity. However, the two reviews differ materially in terms of their analytical scope and intent. Doba et al. employed a predominantly descriptive framework without systematic differentiation of preclinical from clinical evidence levels, without a comparative analysis of SMM versus SAMe, and without addressing the regulatory and translational barriers underlying SMM’s commercial development trajectory. The present review addresses each of these gaps explicitly: (i) evidence-level grading is applied to each pharmacological category and reflected in the summary tables; (ii) SMM is directly compared with SAMe across overlapping therapeutic domains; (iii) translational and regulatory factors limiting SMM’s clinical development are analyzed as a dedicated topic; and (iv) pharmacokinetic data are synthesized with implications for dosage form design. These analytical layers transform the present review from a descriptive inventory of reported effects into a decision-support tool for researchers and developers considering SMM-based clinical or pharmaceutical programs [6].

To date, S-methylmethionine is not registered as a medicinal product in most countries and is predominantly represented in the market as a component of dietary supplements and nutraceuticals. Thus, a trend has been observed toward a shift in its regulatory status from the category of medicinal products to the category of food ingredients or supplements. Registration of SMM as a medicinal product is currently preserved predominantly in certain Asian countries. In Japan, four over-the-counter (OTC) medicinal products and one quasi-drug containing SMM have been registered [78,79,80,81,82]. In the Republic of Korea, six medicinal products with SMM are registered, as well as one pharmaceutical substance (Methylmethionine Sulfonium Chloride, IMCD Korea Co., Ltd., Seoul, South Korea) [83]. In Hong Kong, one medicinal product containing SMM has been registered. Taiwan deserves special attention, where 43 medicinal products with SMM in various dosage forms (granules, powders, injection solutions, and capsules) are registered, with tablet forms being the most widely represented. In addition, one active pharmaceutical ingredient of S-methylmethionine (Methyl Methionine Sulfonium Chloride, Hamari PFST, Ltd., Osaka, Japan) is registered in Taiwan [84]. At the same time, in European Union countries and in the USA, S-methylmethionine is not registered as a medicinal product. In the USA, it is exclusively marketed as a dietary supplement, while in EU countries, it is classified as a food ingredient or component of dietary supplements. In practice, SMM is most often included in specialized nutraceuticals for gastrointestinal support.

Analysis of registered indications demonstrated pronounced homogeneity in the therapeutic positioning of S-methylmethionine. In the overwhelming majority of cases, it is used for peptic ulcer disease of the stomach and duodenum, as well as for acute and chronic gastritis. Additionally, products with S-methylmethionine are widely used for the relief of dyspeptic symptoms, including hyperacidity, pain, stomach cramps, heartburn, bloating, appetite disturbances, nausea, and feelings of fullness. In individual registration dossiers, the spectrum of indications has been expanded to include gastroenteritis, esophagitis, intestinal motility disorders, and functional digestive disorders [78,79,80,81,82,83,84].

Despite the substantial body of experimental data, the level of evidence for certain effects of S-methylmethionine remains limited due to the predominance of preclinical studies and the comparatively small number of contemporary randomized clinical trials. Nevertheless, the consistency of the results obtained, the reproducibility of antioxidant and cytoprotective effects across various models, and the presence of clinical data in gastroenterological practice allow SMM to be considered a biologically active compound with high translational potential. Further research should focus on the standardization of dosage forms, clarification of dose-dependent effects, and well-designed clinical studies to validate its efficacy in new therapeutic indications and regimens of use.

## 6. Conclusions

The available clinical and preclinical data indicate that S-methylmethionine is a multifunctional vitamin-like compound with cytoprotective, antioxidant, and regenerative properties. The most substantiated and clinically confirmed area of its application remains gastroprotection and therapy of inflammatory-destructive diseases of the gastrointestinal tract, where SMM demonstrates the ability to accelerate mucosal healing and increase its resistance to damaging factors.

At the same time, preclinical studies have substantially expanded the pharmacological profile of S-methylmethionine, demonstrating organ-specific protective activity in preclinical models of the nervous system, liver, kidney, lung, skin, and ocular injury—effects that remain to be validated in human studies. These effects are primarily realized through the suppression of oxidative stress, modulation of inflammatory and immune reactions, maintenance of glutathione homeostasis, and activation of intracellular signaling pathways associated with cell survival and proliferation. Additional data indicate the involvement of SMM in the regulation of methylation and cellular regeneration processes, indicating the systemic nature of its action.

Of particular importance in the context of translational prospects is the potential of S-methylmethionine for local application on mucous membranes, including oral cavity tissues. Clinical and experimental data presented in this review indicate its ability to reduce the severity of inflammation and oxidative damage and stimulate reparative processes in periodontal and salivary gland tissues. These properties, combined with a favorable safety profile, substantiate the feasibility of further studies on SMM in local dental dosage forms aimed at the treatment of inflammatory and destructive diseases of the oral mucosa.

Based on the totality of available evidence, the pharmacological effects most ready for Phase II clinical investigation are: (1) SMM’s gastroprotective and mucosal repair activity in NSAID-associated or H. pylori-negative erosive gastropathy, where existing controlled clinical data and validated outcome measures (endoscopic mucosal healing scores, symptom indices) provide a clear biological rationale and trial framework; and (2) SMM’s anti-inflammatory and regenerative action in chronic periodontal disease, where the combination of preliminary clinical evidence and robust preclinical cytoprotective data justifies a well-powered RCT design using established periodontal endpoints. Among the delivery routes, topical and mucosal application—including mucoadhesive gels, films, and rinse formulations for the oral cavity—represents the most pharmaceutically mature approach, supported by advances in SMM stabilization in semi-solid systems and the growing clinical precedent for local mucosal cytoprotection. From a mechanistic perspective, two research priorities stand out: first, the precise contribution of BHMT2-mediated remethylation to SMM’s hepatic and systemic effects across mammalian species relevant to human translation; and second, the role of SMM-driven epigenetic methylation—of both histones and DNA—in its proliferative and regenerative effects, which may account for biological activities extending beyond what methyl donation alone would predict.

## Figures and Tables

**Table 1:** Classification of clinical studies according to intervention type categories (A–D).

Study	Intervention Type
G. Cheney studies [34,35,36]	D
A.S. Salim studies [37,38,39,40]	A
Makhnitska and Babinets [41]	B
Drozdov et al. [42]	A

**Table 2:** Preclinical studies evaluating cytoprotective and antioxidant effects of SMM.

Model	SMM Form/Dose	Intervention Type	Key Assessed Parameters	Main Findings	Mechanistic Notes	Evidence Level/Limitations	Reference
Central Nervous System							
D-galactosamine-induced brain and cerebellum injury in rats	S-methylmethionine sulfonium chloride (pretreatment)	A	GSH levels, TAS, CAT, SOD, ROS, protein oxidation products	Restoration of antioxidant balance; Reduction in oxidative damage to brain and cerebellum tissues	Prevention of GSH depletion and restoration of antioxidant enzyme activity	Level 4/Rat model only; no functional neurological endpoints	Mahmarzayeva et al., 2022 [53]
Pentylenetetrazole-induced brain damage in rats (seizure model)	S-methylmethionine (co-administration)	A	Lipid peroxidation, ROS, NO, oxidative/inflammatory enzymes, total antioxidant capacity	Normalization of biochemical indicators; Neuroprotective effect observed during epileptogenic stress	Realized through antioxidant properties and restoration of antioxidant capacity in brain tissue	Level 4/Single research group *; model has limited translational applicability to human epileptic neuroprotection; no behavioral or functional neurological outcomes assessed; endpoint restricted to biochemical oxidative markers	Bayrak et al., 2022 [54]
Amiodarone-induced brain injury in rats	S-methylmethionine sulfonium chloride (co-administration)	A	GSH, SOD, CAT, Na^+^/K^+^-ATPase, lipid peroxidation, protein carbonylation, MPO, acetylcholinesterase	Reversible restoration of biochemical parameters; Reduction in drug-induced neurotoxicity	Restoration of antioxidant homeostasis and suppression of inflammatory reactions	Level 4/Single research group *; model with limited clinical parallel to therapeutic amiodarone use in humans; no functional neurological or cognitive endpoints; single unreplicated study	Turkyilmaz, 2023 [55]
Ocular							
Valproic acid-induced lens injury in rats	S-methylmethionine sulfonium chloride	A	Lipid peroxidation, aldose reductase, sorbitol dehydrogenase, GSH, SOD, GPx, GR, GST	Prevention of lens injury; Restoration of antioxidant status in lens tissue	Acts as a free radical scavenger and supports the enzymatic antioxidant system	Level 4/Rat ocular model only; no in vivo visual function assessment (ERG or visual acuity); single study from a single group; translational relevance to human drug-induced cataractogenesis requires independent validation	Tunali et al., 2015 [10]
Renal							
Valproate-induced renal damage in rats	S-methylmethionine	A	MDA, xanthine oxidase, GSH, CAT, SOD, TNF-α, IL-1β, MCP-1, TGF-β, Collagen I	Reduced oxidative stress, inflammation, and fibrosis in renal tissue	Combination of antioxidant, anti-inflammatory, and anti-fibrotic mechanisms	Level 4/Single research group *; rat model only; no functional renal endpoints; absence of long-term follow-up; histological confirmation limited	Gezginci-Oktayoglu et al., 2016 [58]
Streptozotocin-induced diabetic nephropathy in mice	S-methylmethionine (dose-dependent)	A	Glomerular hypertrophy, mesangial expansion, fibrosis, macrophage polarization, senescence markers	Amelioration of renal injury; Reduced inflammaging and shifting of macrophages to a reparative phenotype	Inhibition of the ERK/NF-κB signaling pathway to modulate macrophage inflammaging	Level 4/Mouse streptozotocin model; histological outcomes predominate; long-term functional data lacking	Dong et al., 2026 [15]
Hepatic							
Hepatocellular carcinoma induced by DEN and CCl4 (Wistar rats)	S-methylmethionine sulfonium chloride	A	AST, GGT, Albumin, Globulin, TNF-α, iNOS, TGF-β1, Glypican-3, lipid peroxidation	Improved liver function markers; Reduced structural damage and lipid peroxidation; Decreased tumor progression markers	Suppression of inflammatory/immunoregulatory cytokines and modulation of oxidative stress	Level 4/Indirect antitumor effect via antioxidant mechanisms; no direct cytotoxicity data	Abouzed et al., 2021 [57]
Pulmonary							
Valproic acid-induced lung toxicity in rats	S-methylmethionine	A	Lipid peroxidation, antioxidant enzyme activity, structural organization, fibrosis	Restoration of antioxidant protection; Reduction in oxidative damage and pulmonary fibrosis	Amelioration of oxidative stress via modulation of the Nrf2/Keap1 signaling pathway	Level 4/Single research group *; rat model only; no pulmonary function measurements; biochemical endpoints without structural or functional pulmonary correlates; single unreplicated study	Oztay et al., 2020 [59]
Pentylenetetrazole-induced seizures (lung tissue effects) in rats	S-methylmethionine	A	Antioxidant enzymes, NO, inflammatory cell accumulation, collagen fibers	Reduction in oxidative and inflammatory lung damage secondary to seizures	Balancing antioxidant enzymes and reducing oxidative/inflammatory stress in pulmonary tissue	Level 4/Single research group *; pulmonary effects represent an indirect, secondary outcome of a CNS seizure model—not a primary pulmonary injury design; rat model only; no functional respiratory endpoints	Oktay et al., 2018 [9]
Miscellaneous							
Ionizing radiation exposure (general animal model)	S-methylmethionine	A	Biological effect of irradiation, lipid peroxidation, monoamine oxidase activity	Moderate radioprotective activity (15–30%) reduction in biological effect; Dose reduction factor ≈ 1.2)	Antioxidant and membrane-stabilizing nature; Inhibition of lipid peroxidation and MAO activity	Level 4/Old study; moderate radioprotection; modern radiation models absent	Gessler et al., 1996 [60]
Post-radiation effects in experimental animals	S-methylmethionine (in vitamin complexes)	C	Resistance to radiation, survival, and post-radiation metabolic disturbances	Increased resistance to ionizing radiation; Improved survival; reduced metabolic disturbances	Antioxidant activity and synergism with Vitamin E to stabilize cell membranes	Level 4/Old study; SMM administered as part of a vitamin complex, precluding unambiguous attribution of effects; incomplete reporting of species, dose, and survival statistics; not reproduced in modern radiation biology models	Anistratenko, 1992 [61]
Experimental typhoid, dysentery, and staphylococcal infection (guinea pigs)	S-methylmethionine	A	Histomorphological damage to the liver, kidneys, spleen, and lungs	Significant reduction in organ damage; Protective effect in both prophylactic and delayed administration	Cytoprotective rather than immunosuppressive mechanism; avoids the inflammatory-dystrophic changes seen with steroids	Level 4/Very old study; model of uncertain translational relevance to human bacterial disease; no mechanistic data; Soviet-era methodology; results have not been independently replicated in modern in vivo infection models	Kuz’min et al., 1977 [56]

* Studies marked “Single research group” originate from a single Turkish research group (R. Yanardag and colleagues, Istanbul University) and collectively represent the primary preclinical evidence base for SMM’s cytoprotective and antioxidant effects across multiple organ systems. Readers should interpret findings from these studies with awareness of the potential for systematic positive bias inherent in single-laboratory evidence; independent replication is a prerequisite for higher evidence-level classification. Independent replication of these findings using contemporary oxidative stress and inflammatory models—including those employing genetic knockouts or validated pathway inhibitors—is a prerequisite for upgrading the evidence classification of SMM’s cytoprotective effects beyond exploratory.

**Table 3:** Preclinical studies evaluating metabolic effects of S-methylmethionine.

Model	SMM Form/Dose	Intervention Type	Key Assessed Parameters	Main Findings	Mechanistic Notes	Evidence Level/Limitations	Reference
Aminoglycoside-induced nephrotic syndrome (rats)	1000 mg/kg/day, oral	A	Cholesterol Phospholipids Diuresis Proteinuria	↓ Cholesterol ↓ phospholipids Improved nephrotic symptoms	Systemic hypolipidemic effect	Level 4/Old study; rat model only; dose far exceeds clinically used ranges; no functional renal endpoints beyond diuresis and proteinuria; results not independently replicated	Seri et al., 1979 [7]
Hypercholesterolemia models (rats)	Oral	A	Fecal bile acids Neutral sterols	↑ Cholesterol excretion	No effect on absorption or synthesis	Level 4/Old study; mechanistic interpretation inferred indirectly from fecal metabolite data without enzymatic confirmation; rat model only; no human pharmacokinetic data	Seri et al., 1979 [62]
Diet-induced hyperlipidemia (rats)	Oral, dose-dependent	A	LDL HDL	↓ LDL ↑ HDL	Lipoprotein profile modulation	Old study; rat dietary hyperlipidemia model; dose not standardized; no mechanistic data; results from the same single research group as [7,62]—independent replication absent	Seri et al., 1979 [63]
Hyperlipidemia, atheromatosis (rats, rabbits)	Oral	A	Lipids Histopathology	Anti-atherogenic effect	Distinct from clofibrate mechanism	Level 4/Old study; multi-species design without harmonized endpoints; histopathological assessment without blinding reported; single research group; no human translational data	Seri et al., 1980 [64]
Liver microsomes (mice)	Pretreatment	A	7α-hydroxylated cholesterol metabolites	↑ Cholesterol catabolism	Possible CYP7A1 activation	Level 4/Old study; ex vivo microsomal model only—no in vivo confirmation of CYP7A1 activation; mouse model; single study without independent replication; extrapolation to human cholesterol metabolism speculative	Matsuo et al., 1980 [65]
Choline-deficient fatty liver (rats)	Oral	A	Liver morphology Mass	Prevention of steatosis	Opposite effect to methionine	Level 4/Old study; choline-deficient model has limited relevance to contemporary NAFLD/MASLD pathophysiology; comparative methionine arm used without dose-matching; no mechanistic pathway data	Matsuo et al., 1980 [66]
Nutritional deficiency (chickens)	Dietary SMM	A	Growth Metabolic markers	Choline-sparing effect	One-carbon metabolism	Level 4/Avian nutritional model with uncertain translational applicability to human one-carbon metabolism; SMM dose not clinically referenced; endpoint limited to growth performance without molecular mechanistic data	Augspurger et al., 2005 [67]
Metabolic syndrome (rats)	Zn + SMM + vit. C + L-carnitine	C	Fat mass Blood rheology	↓ Visceral fat	Multicomponent formulation	Level 4/Multicomponent formulation precludes attribution of effects to SMM specifically; rat model only; no dose-response data for SMM component; significant confounding by co-administered agents	Matsumoto et al., 2011 [68]
3T3-L1 preadipocytes	In vitro	A	TG accumulation PPARγ AMPK	Anti-adipogenic effect	AMPK activation	Level 4/In vitro model only; 3T3-L1 differentiation does not fully recapitulate human adipogenesis; no in vivo confirmation; apoptosis markers did not reach statistical significance; single study without independent replication	Lee et al., 2012 [69]
High-fat diet (C57BL/6J mice)	Dietary SMM	A	Glucose Insulin HOMA-IR	Improved glucose metabolism	Xenobiotic & circadian gene regulation	Level 4/Mouse model; transcriptomic data without confirmed protein-level or functional metabolic validation; short-term dietary intervention; results require confirmation in human metabolic studies	Egea et al., 2024 [14]

An upward arrow (↑) indicates an increase, whereas a downward arrow (↓) indicates a decrease. Note: The majority of preclinical studies in this table predate 2000 and were conducted by a limited number of independent research groups. Evidence levels are accordingly rated as exploratory/hypothesis-generating. Findings should be interpreted as directional rather than conclusive, pending independent replication in contemporary experimental models.

**Table 4:** Clinical studies evaluating metabolic effects of S-methylmethionine.

Population	SMM Form/Dose	Intervention Type	Duration	Key Assessed Parameters	Main Findings	Notes	Evidence Level/Limitations	Reference
Patients with hypercholesterolemia (*n* = 26)	L-MMSC, 1500 mg/day, oral	A	8 weeks	Total cholesterol triglycerides HDL Lipid ratios	↓ Total cholesterol (−9.7%) ↑ HDL Improved lipid ratios	Stronger effect in hospitalized patients	Level 3/Small sample; no placebo control group; differential effect in hospitalized vs. outpatient subjects unexplained and potentially confounded by diet and concurrent treatment; old study; not replicated in modern lipid-lowering trials	Nakamura et al., 1981 [70]
Healthy subjects and patients with gastroduodenal pathology, postoperative patients	Kobavit (SMM + glutamic acid)	C	Within complex therapy	Glutathione levels GST GR Detoxification markers	Restoration of glutathione system Enhanced detoxification	Combined formulation; SMM contribution indirect	Level 3/Combined formulation—contribution of SMM cannot be isolated from glutamic acid effects; heterogeneous population analyzed without subgroup stratification; no placebo control	Ataliev et al., 2001 [71]
Patients with esophageal and gastric cancer	SMM 1 g × 3/day	A	Postoperative period	Catecholamine methylation Hemodynamics Immune markers	Normalization of methylation processes ↓ hypotension Improved immune parameters	Metabolic correction rather than direct antitumor effect	Level 3/Old study; no randomization or blinding reported; heterogeneous oncological population; hemodynamic and immune endpoints are secondary outcomes without predefined primary endpoint; results not replicated in contemporary oncology studies	Tarutinov et al., 1985 [72]
Patients with lung cancer	SMM (dose not specified)	A	Course therapy	ADA 5′-nucleotidase NK cell activity	↑ NK activity Normalization of adenosine metabolism	Immunometabolic modulation	Level 3/SMM dose not specified; no control arm; adenosine metabolism endpoints are surrogate markers without validated clinical correlates for antitumor activity; Soviet-era methodology; results have not been reproduced in modern immunooncology models	Umanskiĭ et al., 1990 [73]

An upward arrow (↑) indicates an increase, whereas a downward arrow (↓) indicates a decrease. Note: The majority of preclinical studies in this table predate 2000 and were conducted by a limited number of independent research groups. Evidence levels are accordingly rated as exploratory/hypothesis-generating. Findings should be interpreted as directional rather than conclusive pending independent replication in contemporary experimental models.

**Table 5:** SMM evidence levels by indication.

Indication	Evidence Level	Summary of Evidence Base
Gastroprotection	1	RCTs (Salim et al., [37,38,39,40]); controlled clinical studies (Cheney, [34,35,36]); recent prospective data
Periodontal Application	2	One prospective controlled study (Sulym, [45], *n* = 78); preclinical oxidative stress models
Metabolic Effects	3	Uncontrolled clinical observations; single small trial (Nakamura, [70], *n* = 26); no placebo RCT
Neuropsychiatric	3	Single uncontrolled case series (Stoliarov and Mys’ko [50]); no validated instruments; mechanistic rationale indirect
Systemic Anti-Inflammatory	3	Uncontrolled clinical series; preclinical pathway data (ERK/NF-κB)
Radioprotection	4	Preclinical only; no clinical data identified
Dermatoprotective	4	Preclinical only; single research group; no human wound healing RCT
Cytoprotection	4	Preclinical only across CNS, renal, hepatic, pulmonary, and ocular models

## Data Availability

No new data were created or analyzed in this study. Data sharing is not applicable to this article.

## Abbreviations

The following abbreviations are used in this manuscript:

SMM	S-methylmethionine
ERK	extracellular signal-regulated kinase
NF-κB	nuclear factor kappa-light-chain-enhancer of activated B cells
Nrf2	nuclear factor erythroid 2-related factor 2
Keap1	Kelch-like ECH-associated protein 1
MMSC	methylmethionine sulfonium chloride
DL-cysteine	racemic mixture of D- and L-cysteine
GSRS	Gastrointestinal Symptom Rating Scale
SF-36	Short Form-36 (health-related quality of life questionnaire)
L-MMSC	L-form of S-methylmethionine sulfonium chloride
HDL-C	high-density lipoprotein cholesterol
AP	acid phosphatase
cAMP	cyclic adenosine monophosphate
Ca^2+^	calcium ion
BHMT2	betaine-homocysteine S-methyltransferase 2
Hmox1	heme oxygenase-1
PPAR-γ	peroxisome proliferator-activated receptor gamma
C/EBP-α	CCAAT/enhancer-binding protein alpha
AMPK	AMP-activated protein kinase
3T3-L1	murine preadipocyte cell line
HOMA-IR	homeostatic model assessment of insulin resistance
Na^+^/K^+^-ATPase	sodium-potassium adenosine triphosphatase
TNF-α	tumor necrosis factor alpha
iNOS	inducible nitric oxide synthase
TGF-β1	transforming growth factor beta 1
IL-1β	interleukin-1 beta
MCP-1	monocyte chemoattractant protein-1
TGF-β	transforming growth factor beta
ERK1/2	extracellular signal-regulated kinases 1 and 2
UVB	ultraviolet B radiation
UV	ultraviolet radiation
SMMS	S-methylmethionine sulfonium
CYP2A6	cytochrome P450 isoenzyme 2A6
Caco-2	human colorectal adenocarcinoma cell line
HepG2	human hepatocellular carcinoma cell line
MRSA	methicillin-resistant *Staphylococcus aureus*
OTC	over-the-counter
